# Radioligand therapy for primary brain tumors: a PRISMA-based systematic review of meningiomas and gliomas

**DOI:** 10.3389/fmed.2025.1728797

**Published:** 2026-01-23

**Authors:** Ilaria Grassi, Maddalena Sansovini, Federica Matteucci, Irene Marini, Paola Caroli, Monica Celli, Lorenzo Fantini, Virginia Rossetti, Lorena Gurrieri, Nada Riva, Alice Rossi, Ilaria Bronico, Valentina Di Iorio, Anna Sarnelli, Donatella Arpa, Silvia Nicolini

**Affiliations:** 1 Nuclear Medicine Department, IRCCS Istituto Romagnolo per lo Studio dei Tumori "Dino Amadori" (IRST), Meldola, Italy; 2 Medical Oncology Department, IRCCS Istituto Romagnolo per lo Studio dei Tumori "Dino Amadori" (IRST), Meldola, Italy; 3 Radiology Department, IRCCS Istituto Romagnolo per lo Studio dei Tumori "Dino Amadori" (IRST), Meldola, Italy; 4 Radiopharmacy - Pharmacy Department, IRCCS Istituto Romagnolo per lo Studio dei Tumori "Dino Amadori" (IRST), Meldola, Italy; 5 Medical Physics Department, IRCCS Istituto Romagnolo per lo Studio dei Tumori "Dino Amadori" (IRST), Meldola, Italy; 6 Radiotherapy Unit, IRCCS Istituto Romagnolo per lo Studio dei Tumori (IRST) "Dino Amadori", Meldola, Italy

**Keywords:** meningioma peptide receptor radionuclide therapy/PRRT, meningioma radioligand therapy/meningioma RLT, meningioma 90Y/90Y DOTA, meningioma 177Lu/177Lu DOTA, glioma peptide receptor radionuclide therapy, glioma 90Y/90Y DOTA, glioma 177Lu/177Lu DOTA, peptide receptor radionuclide therapy/PRRT

## Abstract

**Introduction:**

There is a critical need for innovative therapies beyond the current standard of care for meningiomas and gliomas. Radioligand therapy (RLT), with its theranostic approach, holds significant promise in this regard. Although several reviews on this topic have been published, none yet have combined the utilization of the Preferred Reporting Items for Systematic Reviews and Meta-Analyses (PRISMA) methodology with the Critical Appraisal Skills Programme (CASP) analysis, along with a dedicated subsection specifically addressing ongoing and completed clinical trials. This review aims to fill this gap in the literature by providing a comprehensive assessment of the current evidence on RLT in these tumors.

**Materials and methods:**

Published studies were searched through PubMed, Scopus, and Web of Science up to 30 April 2025. Only original articles and clinical studies were included. Following a structured selection process, data extraction was performed. Study quality was critically appraised using CASP analyses. For clinical trials, an additional search was conducted on ClinicalTrials.gov beginning on 12 May 2025.

**Results:**

A total of 30 studies were included in the review: 22 on meningiomas (290 patients) and 8 on gliomas (259 patients). For each study, first author, journal, year of publication, somatostatin receptor imaging, study design, radiopharmaceutical used, main topics, response criteria, toxicity assessment, post-therapy scintigraphy, number of patients, WHO grade, demographics, findings and median follow-up were considered. Among clinical trials, 22 were analyzed, including study site, year of first submission, proposed radiopharmaceutical, study type, primary endpoints and status. Efficacy and toxicity data were the primary focus, and the findings were generally encouraging. Studies on RLT in meningiomas was more robust, while in gliomas remained largely experimental. Nevertheless, the authors’ critical appraisal was generally positive. Clinical trials confirmed the more “traditional” nature of research in meningiomas compared to gliomas.

**Conclusion:**

Despite the heterogeneity of the studies, RLT emerges as a promising therapeutic strategy in neuro-oncology. Its theranostic paradigm offers a distinctive advantage, enabling patient selection, treatment personalization, and response monitoring. The development of potentially novel radiopharmaceuticals and the conduct of well-designed multicenter trials with standardized response criteria are needed to further increase the impact and clinical translation of RLT in neuro-oncology.

## Introduction

Meningiomas and gliomas are classified as primary brain tumors, with meningiomas accounting for approximately 30% of cases. According to the World Health Organization (WHO) classification, meningiomas are categorized as typical (grade I, with less aggressive behavior, less risks of recurrence and better prognosis), atypical (grade II), or anaplastic (grade III) (1–3).

Following the latest European Association of Neuro-Oncology (EANO) guidelines, incidental asymptomatic meningiomas should be monitored over time, while for growing or symptomatic ones neurosurgery remains the first-line treatment, when feasible (2). External bean radiation therapy (EBRT) may serve as a complementary treatment or even alternative option to surgery in certain cases (3). Medical treatments— such as bevacizumab, sunitinib, everolimus, temozolomide, irinotecan, imatinib—can be used in cases of recurrent or progressive disease not suitable for surgery or radiotherapy (2).

Gliomas are considered the most common malignant brain tumors. They are classified according to the WHO document by their tumor grade (4) and include different histological subtypes such as astrocytomas, oligodendrogliomas, glioblastomas and ependymomas. These tumors are usually managed through surgical resection, EBRT and chemotherapy, often combined (5), but unfortunately also show a high level of treatment resistance, immune escape and spatiotemporal heterogeneity (6). Multiple factors can explain treatment resistance: first, certain lesions are difficult to reach or resect completely, secondly, and equally important, the presence of the blood–brain barrier (BBB) hinders or postpones the delivery of medications to the tumor (7). Despite efforts to discover new treatments, unfortunately, the prognosis has not much improved and the overall survival (OS) of patients with glioma is still low.

Both meningiomas and gliomas often express somatostatin receptors (SSRs) type 2 (8). Initially, [111In]Pentetreotide scintigraphy (Octreoscan®) was used for imaging these tumors. More recently, hybrid imaging techniques—such as [68Ga]Ga DOTA PET/CT or PET/MRI (DOTA PET)—have been applied in various clinical settings.

Thus, a theranostic approach, based on the use of the same target for both diagnostics and therapy, through peptide receptor radionuclide therapy (PRRT) was proposed for the treatment of meningiomas and gliomas. PRRT is most commonly administered using radiopharmaceuticals such as [^177^ Lu]Lu-DOTA-TOC and [^177^ Lu]Lu-DOTA-TATE (LuPRRT) or [^90^Y]Y-DOTA PRRT (YPRRT). Despite some well-documented differences among the radionuclides employed (9) in terms of type of emission, physical half-life, maximum, and mean *β*-particle energies and penetration depths in soft tissue, LuPRRT and YPRRT similarly combine a molecular vector targeting the SSRs with a beta-emitting isotope. They can be can be delivered systemically by intravenous injection (more frequently), through intratumoral injection, through injection into the tumor resection cavity (10, 11) and finally through intraarterial administration.

The therapeutic landscape has recently expanded from PRRT to the broader concept of radioligand therapy (RLT), with several novel radiopharmaceuticals currently under investigation, such as [177Lu]Lu-prostate specific membrane antigen (PSMA) radioligand therapy (LuPSMA) and the alpha emitters [213Bi] and 225[Ac] to name but a few. The use of alpha particles, on the other hand, should be considered more suitable for smaller lesions, having a shorter travel distance in the substance and higher linear energy transfer (LET) than beta particles.

It is important to note that, at present, meningiomas and gliomas can be treated with RLT only within investigational trials and only in cases of advanced, progressive, or refractory tumors. Findings from these trials have been reported in multiple scientific publications. Consequently, various reviews have been already published on this argument. However, to the best of our knowledge, there are no reviews specifically including at the same time:

- the utilization of the Preferred Reporting Items for Systematic reviews and Meta-Analyses (PRISMA) methodology (12).
- the Critical Appraisal Skills Programme (CASP) analysis.
- the clinical trials currently available or recently completed.

The aim of this publication is to address the current gap in the literature and provide a comprehensive, up-to-date overview of the use of RLT in patients with meningiomas and gliomas, based on available data and clinical trials, and following PRISMA and CASP methodologies.

## Materials and methods

### Information sources and search strategy

#### Published studies

A literature search up to 30 April 2025 was conducted using the PubMed, Scopus, and Web of Science databases. Four authors (M. S., I. G., S. N., and e I. M.) suggested the terms for the research. The following terms were used: meningioma peptide receptor radionuclide therapy/PRRT, meningioma radioligand therapy/meningioma RLT, meningioma 90Y/90Y DOTA, meningioma 177Lu/177Lu DOTA, glioma peptide receptor radionuclide therapy, glioma 90Y/90Y DOTA, and glioma 177Lu/177Lu DOTA. Furthermore, a chronological filter—from 2006 to the present day—was applied to make the research more consistent and aligned with advances in imaging. The search was carried out with the addition of filters, such as English language only and humans subjects only, when possible, that is to say for Pubmed and Scopus. Additionally, filters regarding the type of articles were considered and they were: “Article” for Scopus, while for Pubmed selecting “article” was not possible, so this filter was not applied and the articles were selected later. One reviewer (I. G.) conducted the literature search. Critical appraisal was performed by two reviewers (I. G. and M. S.). and discrepancies, if any, were solved by discussion with the other authors.

Considering the heterogeneity of the studies, a meta-analysis was not performed.

#### Clinical trials

A search was conducted on clinical trials.gov until May 2025. The terms for the research were suggested by four authors (M. S., I. G., S. N., and e I. M.) and included: peptide receptor radionuclide therapy/PRRT, radioligand therapy/RLT, 90Y/90Y DOTA, 177Lu/177Lu DOTA, and meningioma and glioma regarding “Condition/disease.” No additional filters were used. One author (I. G.) conducted the research.

### Selection process

#### Published studies

The inclusion criteria were established by three authors (I. G., M. S., and I. M.) and included original articles and clinical studies, and the studies primarily focussed on therapeutic interventions.

**Figure 1:**
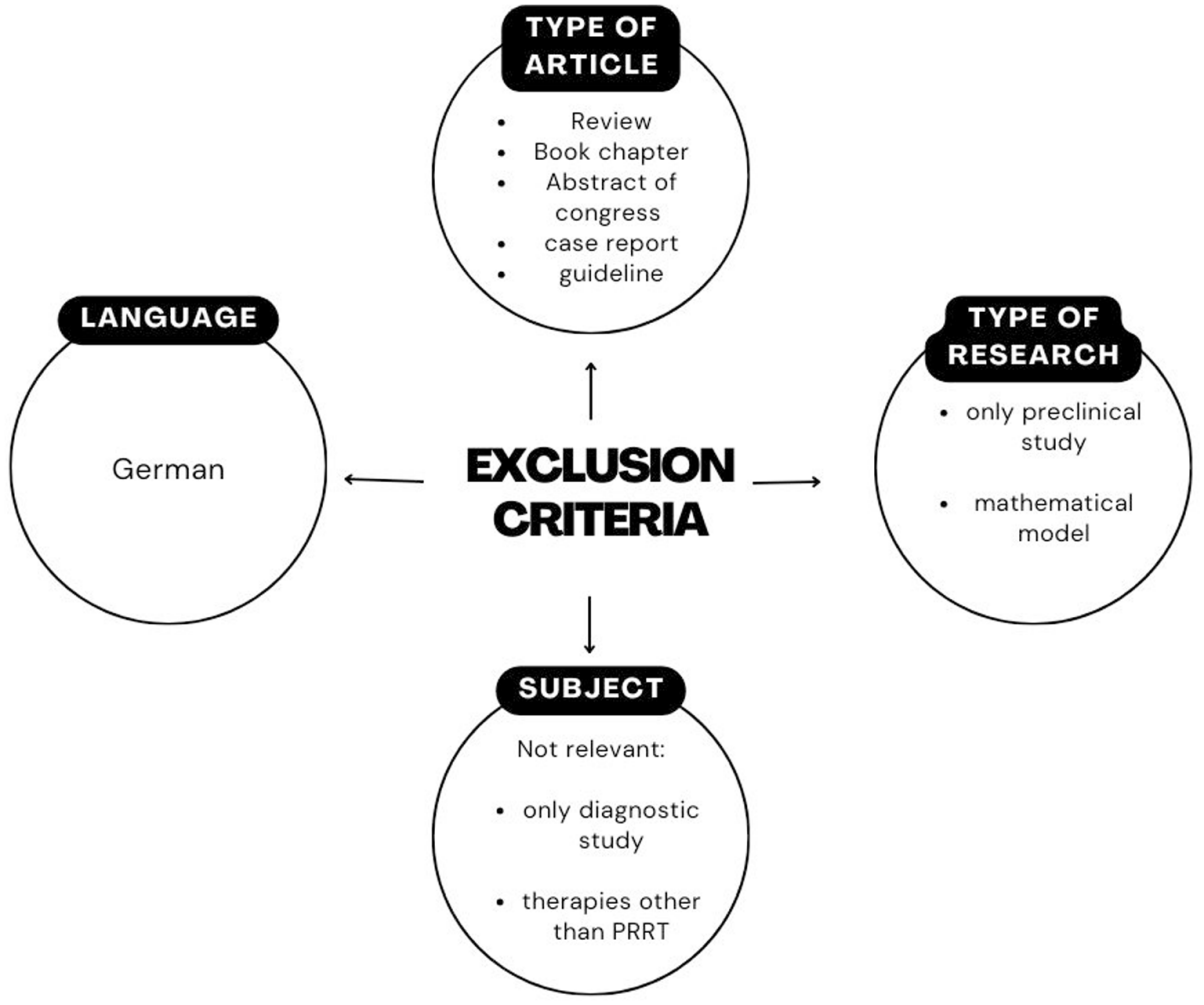
Flowchart illustrating the exclusion criteria applied in the study selection process. Criteria include the type of article, language, type of research and subject relevance. This schematic provides a clear overview of the factors leading to study exclusion.

Exclusion criteria were reported in Figure 1.

One reviewer (I. G.) screened each record and each report retrieved, without the use of automation tools in the process.

#### Clinical trials

Two authors (I. G. and M. S.) carried out the selection process. Trials addressing neoplasms other than meningiomas and gliomas, evaluating agents other than radiopharmaceuticals, or investigating radiopharmaceuticals intended solely for diagnostic purposes were excluded as not relevant to the scope of this review.

### Data extraction

#### Published studies

One reviewer (I. G.) handled the extraction of the data. For any article, the following parameters were taken into account: editorial information (first author, journal, and year of publication), material and methods (study design, radiopharmaceutical used, and treatment schedules), main topics, main findings (efficacy and its endpoints, toxicity and its endpoints, the availability of a post-therapy whole-body scintigraphy WBS, single or serial studies), the number of patients, the WHO tumor grade, the presence or absence of demographic data and the follow-up times.

The studies were analyzed according to the Critical Appraisal Skills Programme (CASP) (https://casp-uk.net/casp-tools-checklists) for qualitative studies, based on ten items and questions. Two reviewers (I. G. and F. M.) filled out the form separately and, in case of discrepancy, a discussion among the authorial group was done.

#### Clinical trials

One reviewer (M. S.) handled the extraction of the data. For any trial, the following parameters were taken into consideration: title, study site(s), year of first submission, investigating radiopharmaceutical, type of study, main endpoints, and current status.

A formal quality assessment was not performed, as results are not yet available.

## Results

### Published studies

#### Search strategy

**Figure 2:**
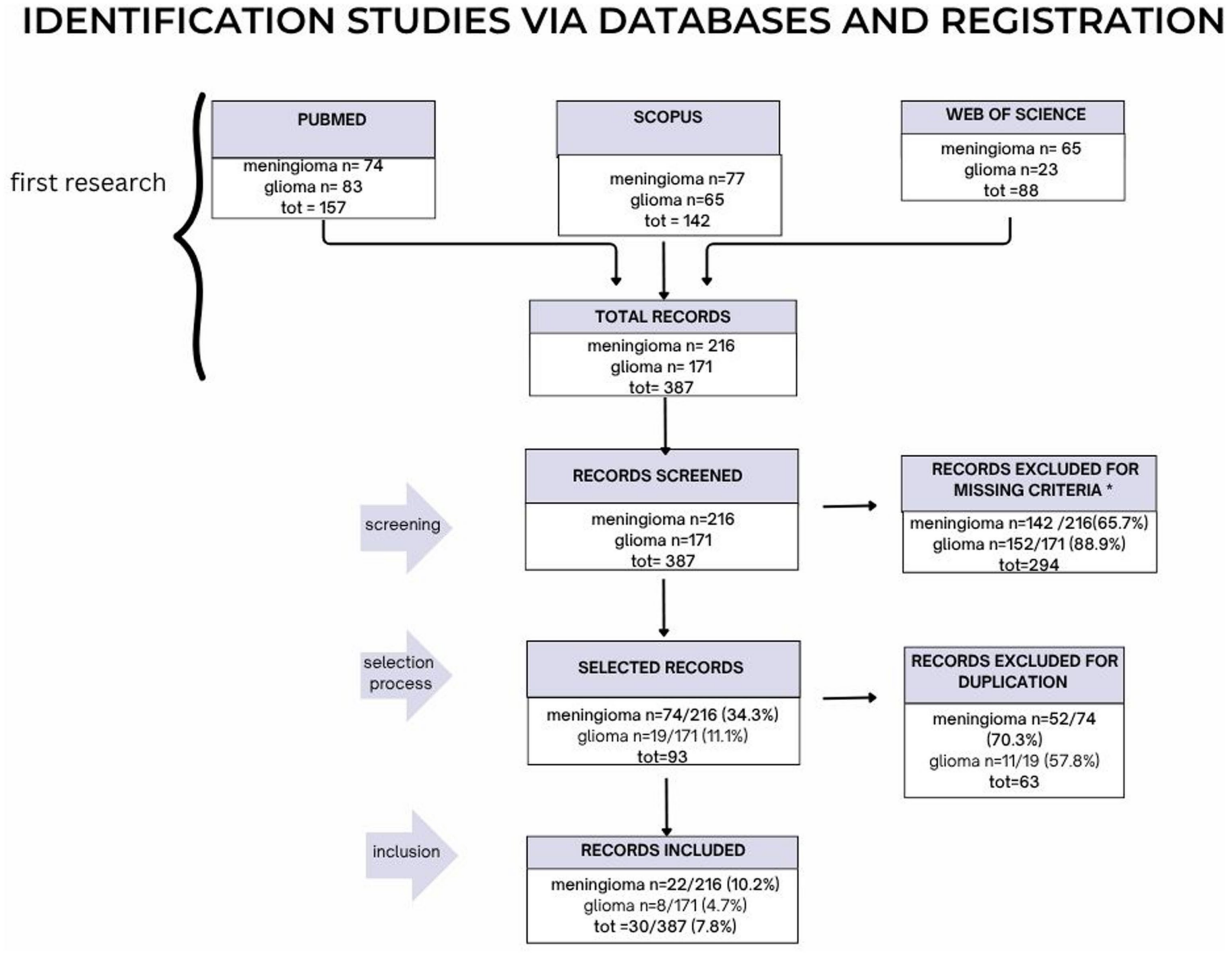
The PRISMA strategy is illustrated here in its parts: screening, selection process and finally inclusion.

The results of PRISMA search strategy is reported in Figure 2. From the systematic literature search, 30 papers were finally selected, including 22 papers dealing with meningiomas and eight with gliomas, for the sum of 290 patients with meningioma and of 259 patients with glioma.

#### Study characteristics

Tables 1, 2 summarize the main findings of the selected studies regarding meningiomas and Tables 3, 4 summarize all the studies regarding gliomas. In Table 5, a comparison is provided between meningioma studies and glioma studies in terms of study design, radiopharmaceuticals, and main findings.

**Table 1:** Overview of published studies on RLT in meningioma (word version).

First author	Journal	Year	SSRs imaging	Study design	RP	Treatment schedules	Main topics	Response criteria	Toxicity measure	WBS	Number of pts	Who grade	Demographics	Main findings	Median FU
Minczeles	European Journal of Nuclear Medicine and Molecular Imaging (EJNMMI)	2022	Octreoscan® and DOTAPET	Retrospective	LuDOTA	Planned TCA of 29.6 GBq, 4 cycles	Efficacy (PFS)and safety	RANO Working Group	CTCAE 4.03	Y (MIRD-based dosimetry)	15	G1, G2, G3, ki 67 10% (4–20).	YES	Efficacy: mOS 13.6 mo TGR declined to 3.1% in surface (*p* = 0.016) and 5.0% in volume. Toxicity: HT: G3 8 pts., G4 in 1	3 mo
Gerster-Gilliéron.	The Journal Of Nuclear Medicine (JNM)	2015	Octreoscan®	Prospective phase 2	YDOTA	7,400 MBq/m2 in 2 fractions.	Efficacy, safety	RECIST	(NCI-CTC V4.0)	Y	15	1 to 3	yes.	Efficacy: mPSF at least 24 mo. Toxicity: HT > G2 in 5 pts. (33.3%), transient G3 lymphocytopenia in 4 pts., transient NT in 2 pts	6 to 12 mo
Minutoli	Cancer biotherapy and radiopharmaceuticals	2014	Octreoscan®	retrospective	[^111^In]In Pentetreotide	2–4 cycles, activity per cycle 1.1–7.4 GBq + YPRRT in 1 pt. and LuPRRT in 1 pt.	Safety, efficacy	SWOG	unknown	N	8	unknown	yes	Toxicity: no G3-G4. Efficacy (DCR): PR in 2 pts., SD in 5 patients, PD in 1 PT	Median of 12 mo
Bartolomei	EJNMMI	2009	Octreoscan®	Prospective	YDOTA	2.5 GBq/cycle every 2/3 months up to a TCA of 15 GBq	Safety and efficacy	SWOG	WHO criteria	Y	29	1 to 3	yes	Efficacy: SD in 19 and PD in10, median OS 40 months. Median TTP: 61 months in grade 1 and 13 in grade 2 and 3. No NT, no G3 or G4 other toxicity.	Last update oct 2007
Kreissl	Radioation oncology	2012	DOTAPET	Pilot trial	LuDOTA	7.4 ± 0.3 GBq	Safeyt and efficacy of PRRT + EBRT	ce MRI (volume changes of 25% to define progression or response and DOTAPET)	CTCAE 4.0	Y	10	1 and 2	yes	Efficacy: SD in 8/10 pts. 1/10 CR No chronic effects >G 1	Median of 13.4 mo
Hanscheid	JNM	2017	Octreoscan®	retrospective	LuDOTA	22; 5.3–8.1 GBq	dosimetry	NA	NA	Y OLINDA /EXM based dosimetry	29, 8 with meningioma	–	no	A single post-therapeutic measurement (4 d after injection) by SPECT/CT could be used be used to estimate the absorbed with minor additional resources and effort.	–
Vonken	JNM	2021	DOTAPET	retrospective	LuDOTA (high affinity)	7.400 MBq/cycle for 4 cycles,the first one intravenous, the subsequent ones intraarterially.	intrapatient comparison betwwen intravenous and intraarterial treatment (safety and efficacy)	RANO Working Group 2019	CTCAE 5.0	Y	4	2 and 3	yes	Toxicity: 1 pt. with G3 HT (Leukopenia). Efficacy: 3 pts. completed treatment: 1 was PR, 2 SD. They all Improved clinical conditions	Median 1.7 y
Amerein	JNM	2024	DOTAPET	retrospective	LuDOTA (high affinity)	1–4 cycles with mean activity/cycle of 7.428 MBq	Safety and efficacy of intraarterial PRRT	RANO Working Group	CTCAE 5.0	Y	13	1–3	yes	HT: 3 pts. with G3 thrombocypenia, 5 with G3 lymphocytopenia and 1 with G4 lymphocytopenia. 1 pt. had transient G3-G4, RN (probably not reated to PRRT), 1 pt. with local necrosis (probably related to angiography). Efficacy. 1/10 CR, 1/10 PR, 8/10 SD, 9/13 CI m PFS of 18 mo	Up to 43 mo
Hartrampf	Clinical and Translational Radiation Oncology	2020	DOTAPET	retrospective	LuDOTA	7–7.9 GBq/cycle	Efficacy and safety of PRRT+EBRT	RANO Working Group	CTCAE 5.0	Y	10	2 and 3	yes	Toxicity: no severe acute or chronic toxicity. Efficacy: SD in 7/10; mPFS ranging from 13.8 to 107.7 months, OS from 38.2 to 111.4	Median of 105.0 mo
Kertels	Clinical nuclear medicine	2021	DOTAPET	retrospective	LuDOTA	Median of 4 cycles. TCA 9.6–39.5 GBq	PRRT in neurofibromatosis and meningioma, efficacy and safety	RANO Working Group	CTCAE 5.0	Y	11	1–3	yes	HT: 5 pts. anemia>G3, 7 pts. trombocytopenia >G3, 9 pts. leukopemia >G3. Efficacy: mPSF 12 mo, OS 37 mo. SD in 6 pts	Median 27 mo
Kletting	JNM	2016	–	retrospective	YDOTA	unnkown	Dosimetry Optimizing YPRRT	NA	NA	Y OLINDA /EXM based dosimetry	4	unknown	+/−	Optimal activity of 4.2 ± 1.8 GBq for meningioma	–
van Essen	JNM	2006	Octreoscan®	Prospective?	LuDOTA	7.4 Gbq/cycle, TCA of 22.2–29.6	Safety and efficacy of PRRT in different tumors	recist	WHO	Y	22, 5 with meningioma	unknown	yes	Efficacy: 4 PD, 1 SD. Toxicity for pts. with meningioma unclear	unclear
Graillon	Journal of Neuro-Oncology	2013	DOTAPET	retrospective	LuDOTA	Lutathera schedule	Impact of PRRT on 3DVGR	RANO Working Group	no	N	8	1 and 2	yes	3DVGR significantly decreased at 3, 6, and 12.	18 mo
Hänscheid	EJNMMI	2012	DOTAPET	Pilot trial	LuDOTA	7.4 GBq	Correlation between tumour uptake of PRRT and PET pre-PRRT	NA	no	Y	11	≥2	yes	Strong correlation between SUV and TR	3 mo after PRRT
Eigler	JNM	2024	DOTAPET	Prospective phase 0	LuDOTA	6.9–7.3 GBq of [177 Lu]Lu-DOTATOC followed by 3.3–4.9 GBq of [177 Lu]Lu-DOTA-JR11 at an interval of 10 ± 1wk	Comparison between [^177^Lu]Lu-DOTA-JR11 And LuPRRT in terms of therapeutic index (tumor–to– bone marrow and tumor-to-kidney absorbed-dose ratios)	progressive disease was defined as at least a 40% increase of meningioma volume or new lesions; and stable disease was definedaslessthana 40% increase in volume	CTCAE 5.0	Y OLINDA /EXM based dosimetry	6	1–3	yes	Median absorbed dose of 1 cycle: 3.4 Gy for LuPRRT and 11.5 Gy for DOTA-JR11. Toxicity: 2 pts. with G3 lymphopenia and 1 pt. with G3 lymphopenia and neutropenia after DOTA-JR11. Efficacy DRC: 83%	18 mo
Puranik	Neurology India	2024	DOTAPET	Retrospective	LuDOTA	First cycle intravenous, 4 pts. continued with intraarterial 7.4 GBq/cycle every 8–12-weeks	Efficacy and safety of intavenous and subsequent intrarterial administration of PRRT	RANO Working Group	Unknown	Y OLINDA /EXM based dosimetry	8	1–3	yes	Efficacy: median TTP of 8.9 months, PD in 2 pts., PR in 2 pts., 4 pts. in SD. Toxicity: unclear	12 mo
Severi	JNM	2024	Octreoscan® and DOTAPET	Prospective	LuDOTA and YDOTA	TCA of 11.1 for 90Y and of 22 GBq for Lu, divided into 4 to 5 cycles every 5–8 wks TCA for rechallenge:13 GBq of 177 Lu DOTATATE	Efficacy and safety of PRRT, rechallenge	RANO Working Group	CTCAE v 4.0 and 5	NA	42	1–3	yes	Efficacy: DCR of 57%, mPFS of 16 mo, mOS 36 mo. Toxicity: G3 platelets toxicity in 1 pt. No symptomatic worsening of conditions. Efficacy: For rechallenge: mPFS of 6.5 mo and mOS of 17 mo	63 mo 75.8 for rechallenge
Reuvers	Cancers	2024	–	unknown	LuDOTA	NA	*In vitro* model for PRRT (spheroids)	NA	NA	NA	16	1 and 2	yes	PRRT induced DNA damage, correlated with SSTR2-expression.	NA
Dubois	Cancer biotherapy and radiopharmaceuticals	2024	DOTAPET	restrospective	LuDOTA	Luthathera schedule	Factors associated with safety	NA	CTCEA 5.0	N	46 but 40 evaluable, 5 with meningioma	unknown	no	Toxicity: 14 pts. with G3 or higher HT with a single parameter; 3 pts. with 2 parameters; 4 pts. with 3 parameters; and 2 ptswith 4 parameters. 1 pt. had G3 hepatic cytolysis. Risk factors not considered separately for meningioma	for 6 mo after the last injection.
Salgues	Current oncology	2022	DOTAPET	restrospective	LuDOTA	3,200–7,400 MBq for 4 cycles every 8/9 wks	Safety and efficacy	RANO Working Group	CTCAE v6.0	Y	8	2	yes	Toxicity: frequent SE transient G1 HT. 3 pts. G3 lymphocytopenia. Efficacy: 5/6 pts. with SD at 12 months. PFS at 6mo was 85.7% and PFS at 12mo was 66.7%	unknown
Seystahal.	Neuro-Oncology	2016	DOTAPET	retrospective	LuDOTAand YDOTA	median of 3 cycles, dose/cycle 3,400–7,400 MBq	Safety and efficacy	RANO Working Group	CTCAE v 4.0	N	20	1–3	yes	Efficacy: SD in 10/20, mPFS and PFS at 6 mo stratified according to grade, as such as mOS. Ga DOTA uptake was linked to MRI imaging	20 mo
Marincek	JNM	2015	Octreoscan®	Prospective phase 2	LuDOTA and YDOT.	Cycles repeated until tumor progression or permanent toxicity occurred	Safety and efficacy	RECIST 1.1	CTCAE v 3.0	Y	74 treatments, 34 pts. evaluable	unknown	yes	Efficacy: SD in 23 pts. Relevant HT in 3 pts. and severe RT in 1 pt. MS of 8.6 y from recruitment. SD and and high tumor uptake associated with longer survival.	21.8 mo

This table provides a comprehensive summary of clinical studies, including in particular imaging modalities, study design, primary objectives, post therapy WBS and/or dosimetric evaluations, patient and tumor characteristics and outcomes in terms of efficacy and safety. Both retrospective and prospective experiences are included, highlighting heterogeneous protocols and patient populations while illustrating feasibility, efficacy and safety of RLT in progressive or pretreated meningiomas.

RP: radiopharmaceutical; FU: follow-up; Mo: months; Octreoscan®: [^111^In]In-DTPA-octreotide scintigraphy; DOTA PET: [^68^ Ga]Ga DOTA PET; LuDOTA: [^177^Lu]Lu DOTA-TATE or [^177^Lu]Lu-DOTA-TOC; YDOTA: [^90^ Y]Y DOTA-TOC; HT: hematological toxicity; NT: neurological toxicity; RN: renal toxicity; DCR: disease control rate; PR: partial responders; SD: stable disease; PD: progression disease; CR: complete responders; TTP: time to progression; CI: clinical improvement; 3DVGR: MRI three-dimensional volume growth rate; TR: PRRT radionuclide tumour retention; [^177^Lu]Lu-DOTA-JR11: LuJR11; SE: side effect; MS: Mean survival; Y: yes; N: no; WBS: Post-therapy whole-body scintigraphy (single or serial studies).

**Table 2:** Overview of published studies on RLT in meningioma (excel version).

First author	Journal	Year	SSRs imaging	Study design	RP	Treatment schedules	Main topics	Response criteria	Toxicity	WBS	Number of pts	Who grade	Demographics	Main findings	Median FU
Minczeles	EJNMMI	2022	Octreoscan® and DOTAPET	Retrospective	LuDOTA	Planned TCA of 29.6 GBq, 4 cycles	Efficacy (PFS)and safety	RANO Working Group	CTCAE 4.03	Y	15	G1, G2, G3, ki 67 10% (4–20).	YES	Efficacy: mOS 13.6 mo	3 mo
Gerster-Gilliéron.	The Journal Of Nuclear Medicine	2015	Octreoscan®	Prospective phase 2	YDOTA	7,400 MBq/m2 in 2 fractions.	Efficacy, safety	RECIST	(NCI-CTC V4.0)	Y	15	1 to 3	Yes.	Efficacy: mPSF at least 24 mo.	6 to 12 mo
Minutoli	Cancer biotherapy and radiopharmaceuticals	2014	Octreoscan®	Retrospective	[^111^In]In Pentetreotide	2–4 cycles, activity per cycle 1.1–7.4 GBq + YPRRT in 1 pt. and LuPRRT in 1 pt	Safety, efficacy	SWOG	unknown	N	8	unknown	yes	Toxicity: no G3-G4. Efficacy (DCR): PR in 2 pts., SD in 5 patients, PD in 1 PT	Median of 12 mo
Bartolomei	EJNMMI	2009	Octreoscan®	Prospectic	YDOTA	2.5 GBq/cycle every 2/3 months up to a TCA of 15 GBq	Safety and efficacy	SWOG	WHO criteria	Y	29	1 to 3	yes	Efficacy: SD in 19 and PD in10, median OS 40 months. Median TTP: 61 months in grade 1 and 13 in grade 2 and 3. No NT, no G3 or G4 other toxicity	Last update oct 2007
Kreissl	Radioation oncology	2012	DOTAPET	Pilot trial	LuDOTA	7.4 ± 0.3 GBq	Safeyt and efficacy of PRRT + EBRT	ce MRI (volume changes of 25% to define progression or response and DOTAPET)	CTCAE 4.0	Y	10	1 and 2	yes	Efficacy: SD in 8/10	Median of 13.4 mo
Hanscheid	JNM	2017	Octreoscan®	retrospective	LuDOTA	22; 5.3–8.1 GBq	dosimetry	NA	NA	Y OLINDA/EXM based dosimetry	29, 8 with meningioma	–	no	A single post-therapeutic measurement could estimate the absorbed dose	–
Vonken	JNM	2021	DOTAPET	retrospective	LuDOTA (high affinity)	7.400 MBq/cycle for 4 cycles,the first one intravenous, the subsequent ones intraarterially.	intrapatient comparison betwwen intravenous and intraarterial treatment (safety and efficacy)	RANO Working Group 2019	CTCAE 5.0	Y	4	2 and 3	yes	Toxicity:1 pt. with G3 HT (Leukopenia). Efficacy: 3 pts. completed treatment: 1 was PR, 2 SD. They all Improved clinical conditions	Median 1.7 y
Amerein	JNM	2024	DOTAPET	retrospective	LuDOTA (high affinity)	1–4 cycles with mean activity/cycle of 7.428 MBq	Safety and efficacy of intraarterial PRRT	RANO Working Group	CTCAE 5.0	Y	13	45717.00	yes	HT: 3 pts. with G3 thrombocypenia, 5 with G3 lymphocytopenia and 1 with G4 lymphocytopenia. RN: 1 pt. with G3-G4, 1 pt. with local necrosis. Efficacy: 1/10 CR, 1/10 PR, 8/10 SD, 9/13 CI m PFS of 18 mo	Up to 43 mo
Hartrampf	Clinical and Translational Radiation Oncology	2020	DOTAPET	retrospective	LuDOTA	7–7.9 GBq/cycle	Efficacy and safety of PRRT+EBRT	RANO Working Group	CTCAE 5.0	Y	10	2 and 3	yes	no toxicities; mPFS 13.8–107.7 mo, OS 38.2 to 111.4	Median of 105.0 mo
Kertels	Clinical nuclear medicine	2021	DOTAPET	retrospective	LuDOTA	Median of 4 cycles. TCA 9.6–39.5 GBq	PRRT in neurofibromatosis and meningioma, efficacy and safety	RANO Working Group	CTCAE 5.0	Y	11	45717.00	yes	HT: 5 pts. anemia>G3, 7 pts. trombocytopenia >G3, 9 pts. leukopemia >G3. Efficacy: mPSF 12 mo, OS 37 mo. SD in 6 pts.	Median 27 mo
Kletting	JNM	2016	–	retrospective	YDOTA	unnkown	Dosimetry Optimizing YPRRT	NA	NA	Y	4	unknown	+/−	Optimal activity of 4.2 ± 1.8 GBq for meningioma	–
van Essen	JNM	2006	Octreoscan®	Prospective?	LuDOTA	7.4 Gbq/cycle, TCA of 22.2–29.6	Safety and efficacy of PRRT in different tumors	recist	WHO	Y	22, 5 with meningioma	unknown	yes	Efficacy: 4 PD, 1 SD. Toxicity uncleae	unclear
Graillon	Journal of Neuro-Oncology	2013	DOTAPET	retrospective	LuDOTA	Lutathera schedule	Impact of PRRT on 3DVGR	RANO Working Group	no	N	8	1 and 2	yes	3DVGR significantly decreased at 3, 6, and 12.	18 mo
Hänscheid	EJNMMI	2012	DOTAPET	Pilot trial	LuDOTA	7.4 GBq	Correlation between tumour uptake of PRRT and PET pre-PRRT	NA	no	Y	11	≥2	yes	Strong correlation between TR ansd SUV	3 mo after PRRT
Eigler	JNM	2024	DOTAPET	prospective phase 0	LuDOTA	6.9–7.3 GBq Lu-DOTATOC followed by 3.3–4.9 GBq of [177 Lu]Lu-DOTA-JR11	comparison between LuDOTA JR11 and LuPRRT	PD atleast a 40% increase volume or new lesions; SD less than 40% increase i	CTCAE 5.0	Y OLINDA/EXM based dosimetry	6	from 1 to 3	yes	Toxicity: 2 pts. with G3 lymphopenia and 1 pt. with G3 lymphopenia and neutropenia after DOTA-JR11. Efficacy DRC: 83%	18 mo
Puranik.	Neurology India	2024	DOTAPET	Retrospective	LuDOTA	First cycle intravenous, 4 pts. continued with intraarterial 7.4 GBq/cycle every 8–12 weeks	Efficacy and safety of intavenous and subsequent intrarterial administration of PRRT	RANO Working Group	Unknown	Y OLINDA/EXM based dosimetry	8	from 1 to 3	yes	Efficacy: median TTP of 8.9 months, PD in 2 pts., PR in 2 pts., 4 pts. in SD Toxicity: unclear	12 mo
Severi	JNM	2024	Octreoscan® and DOTAPET	Prospective	LuDOTA and YDOTA	TCA of 11.1 for 90Y and of 22 GBq for Lu, divided into 4 to 5 cycles every 5–8 wks	Efficacy and safety of PRRT, rechallenge	RANO Working Group	CTCAE 4.0 and 5	NA	42	from 1 to 3	yes	Efficacy: DCR of 57%, mPFS of 16 mo, mOS 36 mo. Toxicity: G3 platelets toxicity in 1 pt. No symptomatic worsening of conditions. Efficacy: For rechallenge: mPFS of 6.5 mo and mOS of 17 mo	63 mo 75.8 for rechallenge
Reuvers	Cancers	2024	-	unknown	LuDOTA	NA	In vitro model for PRRT (spheroids)	NA	NA	NA	16	1 and 2	yes	PRRT induced DNA damage, correlated with SSR expression	NA
Dubois	Cancer biotherapy and radiopharmaceuticals	2024	DOTAPET	restrospective	LuDOTA	Luthathera schedule	Factors associated with safety	NA	CTCEA 5.0	N	46 but 40 evaluable, 5 with meningioma	unknown	no	Toxicity: 14 pts. with G3 or higher HT with a single parameter;3 pts. with 2 parameters; 4 pts. with 3 parameters; and 2 pts. with 4 parameters. 1 pt. had G3 hepatic cytolysis. Risk factors not considered separately for meningioma	for 6 mo after the last injection.
Salgues	Current oncology	2022	DOTAPET	restrospective	LuDOTA	3,200–7,400 MBq for 4 cycles every 8/9 wks	Safety and efficacy	RANO Working Group	CTCAE v6.0	Y	8	2.00	yes	Toxicity: frequent SE transient G1 HT. 3 pts. G3 lymphocytopenia. Efficacy: 5/6 pts. with SD at 12 months. PFS at 6mo was 85.7% and PFS at 12mo was 66,7%	unknown
Seystahal	Neuro-Oncology	2016	DOTAPET	retrospective	LuDOTAand YDOTA	median of 3 cycles, dose/cycle 3,400–7,400 MBq	Safety and efficacy	RANO Working Group	CTCAE v 4.0	N	20	from 1 to 3	yes	Efficacy: SD in 10/20, mPFS and PFS at 6 mo stratified according to grade, as such as mOS. Ga DOTA uptake was linked to MRI	20 mo
Marincek	JNM	2015	Octreoscan®	Prospective phase 2	LuDOTA and YDOT.	Cycles repeated until tumor progressionor permanent toxicity occurred	Safety and efficacy	RECIST 1.1	CTCAE v 3.0	Y	74 treatments, 34 pts. evaluable	unknown	yes	Efficacy: SD in 23 pts. Relevant HT in 3 pts. and severe RT in 1 pt. MS of 8.6 y from recruitment. SD and high tumor uptake associated with longer survival.	21.8 mo

This table provides a comprehensive summary of clinical studies, including in particular imaging modalities, study design, primary objectives, post therapy WBS and/or dosimetric evaluations, patient and tumor characteristics and outcomes in terms of efficacy and safety. Both retrospective and prospective experiences are included, highlighting heterogeneous protocols and patient populations while illustrating feasibility, efficacy and safety of RLT in progressive or pretreated meningiomas.

RP: radiopharmaceutical; FU: follow-up; Mo: months; Octreoscan®: [^111^In]In-DTPA-octreotide scintigraphy; DOTA PET: [^68^ Ga]Ga DOTA PET; LuDOTA: [^177^Lu]Lu DOTA-TATE or [^177^Lu]Lu-DOTA-TOC; YDOTA: [^90^ Y]Y DOTA-TOC; HT: hematological toxicity; NT: neurological toxicity; RN: renal toxicity; DCR: disease control rate; PR: partial responders; SD: stable disease; PD: progression disease; CR: complete responders; TTP: time to progression; CI: clinical improvement; 3DVGR: MRI three-dimensional volume growth rate; TR: PRRT radionuclide tumour retention; [^177^Lu]Lu-DOTA-JR11: LuJR11; SE: side effect; MS: Mean survival; Y: yes; N: no; WBS: Post-therapy whole-body scintigraphy (single or serial studies).

**Table 3:** Overview of published studies on RLT in gliomas (word version).

First author	journal	Year	Study design	SSRimaging	RP	Treatment schedules	Main topics	Response criteria	Toxicity measure	WBS	Number of pts	WHO grade	Demographics	Main findings	Median FU
Li	Journal of neurosurgery Online	2010	Prospective phase 2	NA	^125^I-mAb425	1.8 GBq over a course of 3 weekly administration	RIT:efficacy and safety	Kaplan–Meier curves	CTCAE v.2010	no	192 treated with RIT, 60 also with temozolomide (RIT + TMZ)	Grade 4 astrocytoma	yes	mOS 15.7 mo, 1 y survival 62.5%, 2 y survival 25.5%, better in RIT. 7 (3.6%) pts. with acute SE mostly G1-G2 (flushing, nausea, hypotension, skin irritation at the injection site). 4 patients became HAMA positive No Grade 3 or 4 toxicities. TMZ + RIT groups no major toxicities	unclear
Keinfel	EJNMMI	2007	prospective	–	[⁹⁰Y]Y-DOTA GAsubstance P	370–3.330 MBq	dosimetry (using 2 MBq of [^111^ In]In-substance P)	NA	NA	Yes	12	4 four glioblastoma(grade 4), 2 anaplastic gliomas (grade 3) and 6 low-grade astrocytomas (grade 2I).	Yes (not detailed)	Very good agreement between pre- and post-therapeutic dosimetry Good correspondence between the pretherapeutic test injection and the dose deposition	NA
Krolicki	International journal of molecular science	2023	Pivotal study	^−^	[^213^Bi] Bi/^225^[Ac] Ac DOTA-substance P	2–2.5 GBq of [^213^Bi] Bi DOTA-substance P or 17–35 MBq ^225^[Ac] Ac DOTA-substance P, intratumoral injection	TAT: efficacy and safety	NA	unknown	no	11	oligodendroglioma grade 2 and astrocytoma grade 2	yes	RFS of 2–16 ys in astrocytoma (8 pts) and of 4–24 ys in oligodendroglioma (3pts) Low neurotoxicity	1–24 years (median 10)
Cordier-Forrer Kneifel	Journal of Neurooncol	2009	Prospective phase 1	–	[⁹⁰Y]Y-DOTA DOTAGA–substance P	Intratumoral injection of [^90^Y]Y-DOTAGA–substance P at weekly intervals. TCA: 120–345 mCi with dose escalation	Feasibility and safety	NA	CTCAE v 2.0	Yes + Test injection before surgerywith [^111^In]In-DOTAGA-SP	17	Unknown	yes	No relevant SE. No increased intracranial pressure. During surgery better resectability and reduced intraoperative bleeding. 1 pt. with epidural and subdural abscess, 1 pt. with local fistula, 1 pt. with acute intracerebral hemorrhage for tumor regrowth.	unclear
Truckenmueller	Frontiers in oncology	2022	retrospective cohort study,	PSMA PET	LuPSMA	6.03 (5.74–6.10) GBq/cycle for 2 cycle every 9–11 wks	correlation between [68 Ga]Ga-PSMA uptake and histological PSMA expression, TBR and rLuPSMA	NA	CTCAE v 5.0	yes	3	1 glioblastoma and 2 astrocytoma	yes	20 pts. included mSUVmax 4.5 (3.7–6.2) High TBR correlated with increased endothelial PSMA expression. Only 3 pts. had TBRmax>1.0 and qualified for LuPSMA RLT. No SE.observed No efficay data are given	maximum 15 weeks
Heute	JNM	2010	prospective	DOTA PET	YDOTA	TCA 1.7–2.2 GBq in 3 or 4 cycles locally injected into a previously implanted catheter system every 3 mo	Efficacy and safety	FUI: ceMRI DOTA PET, FDG PET, FET PET	unknown	yes	3	Grade 4 glioblastoma	yes	Treatment successful in all 3 patients, with only minor SE, such as epileptic seizure, transient, mild headache 1 pt. CR, 2 pts. PR clinical improvement and improved QOL	Unclear, probably 4 years
Cordier Forrer Bruchertseifer	EJNMMI	2009	Pilot trial	^−^	^213^Bi-DOTA -substance P:	Intratumoral injection, 3–5 injections over 2 days. 4 pts. with TCA 1.07–2.00 GBq, 1 pt. TCA 7.36 GBq divided into 4 cycles	Feasibility and safety	MRI	CTCAE v2.0	Yes (with blood sampling)	5	glioma (grade 2–4)	yes	feasible and tolerated without additional neurological deficit. No relevant AE. Possible radiation induced necrosis	unclear
Nemati	CNM	2021	prospective	Octreoscan® and DOTA PET	LuDOTA	1–4 cycles every 1–2 mo, TCA 3.7–26.9 GBq	Efficacy, safety, qol	RANO, Karnoski performance status	CTCAE 4.03	yes	16	3 and 4	yes	CR in 2 pts., PR in 5 pts., SD in 3 pts., PD in 6 pts. No relevant SE. No significant improvement of qol	1–26 months

This table provides a comprehensive summary of clinical studies, including in particular imaging modalities, study design, primary objectives, post therapy WBS and/or dosimetric evaluations, patient and tumor characteristics and outcomes in terms of efficacy and safety. Both prospective and retrospective studies, as well as pilot and pivotal trials, are included. The table highlights feasibility, tolerability and potential clinical benefit of RLT in malignant gliomas, with generally low rates of severe adverse events.

RP: radiopharmaceutical; FU: follow-up; Mo: months; Octreoscan®: [^111^In]In-DTPA-octreotide scintigraphy; DOTA PET: [^68^ Ga]Ga DOTA PET; LuDOTA: [^177^Lu]Lu DOTA-TATE or [^177^Lu]Lu-DOTA-TOC; YDOTA: [^90^ Y]Y DOTA-TOC; HT: hematological toxicity; NT: neurological toxicity; RN: renal toxicity; DCR: disease control rate; PR: partial responders; SD: stable disease; PD: progression disease; CR: complete responders; TTP: time to progression; CI: clinical improvement; 3DVGR: MRI three-dimensional volume growth rate; TR: PRRT radionuclide tumour retention; [^177^Lu]Lu-DOTA-JR11: LuJR11; SE: side effect; MS: Mean survival; Y: yes; N: no; WBS: Post-therapy whole-body scintigraphy (single or serial studies).

**Table 4:** Overview of published studies on RLT in gliomas (excel version).

First author	Journal	Year	Study design	SSRimaging	RP	Treatment schedules	Main topics	Response criteria	Toxicity measure	WBS	Number of pts	WHO grade	Demographics	Main findings	Median FU
Li	Journal of neurosurgery Online	2010	Prospective phase 2	NA	^125^I-mAb425	1.8 GBq over a course of 3 weekly administration	RIT:efficacy and safety	Kaplan–Meier curves	CTCAE v.2010	no	192 treated with RIT, 60 also with temozolomide (RIT + TMZ)	Grade 4 astrocytoma	yes	mOS 15.7 mo, 1 y survival 62.5%, 2 y survival 25.5%, better in RIT.7 (3.6%) pts. with acute SE mostly G1-G2 (flushing, nausea, hypotension, skin irritation at the injection site). 4 patients became HAMA positive No Grade 3 or 4 toxicities. TMZ + RIT groups no major toxicities	unclear
Keinfel	EJNMMI	2007	prospective	–	[⁹⁰Y]Y-DOTA GAsubstance P	370–3.330 MBq	dosimetry (using 2 MBq of [111 In]In-substance P)	NA	NA	Yes	12	4 four glioblastoma (grade 4), 2 anaplastic gliomas (grade 3) and 6 low-grade astrocytomas (grade 2I).	Yes (not detailed)	Very good agreement between pre- and post-therapeutic dosimetry Good correspondence between the pretherapeutic test injection and the dose deposition	NA
Krolicki	International journal of molecular science	2023	Pivotal study	^–^	[^213^Bi] Bi/^225^[Ac] Ac DOTA-substance P	2–2.5 GBq of [^213^Bi] Bi DOTA-substance P or 17–35 MBq ^225^[Ac] Ac DOTA-substance P, intratumoral injection	TAT: efficacy and safety	NA	unknown	no	11	oligodendroglioma grade 2 and astrocytoma grade 2	yes	RFS of 2–16 ys in astrocytoma (8 pts) and of 4–24 ys in oligodendroglioma (3pts) Low neurotoxicity	1–24 years (median 10)
Cordier-Forrer Kneifel	Journal of Neurooncol	2009	Prospective phase 1	–	[⁹⁰Y]Y-DOTA DOTAGA–substance P	Intratumoral injection of [90Y]Y-DOTAGA–substance P atweekly intervals. TCA: 120–345 mCi with dose escalation	Feasibility and safety	NA	CTCAE v 2.0	Yes	17	Unknown	yes	No relevant SE. No increased intracranial pressure. During surgery better resectability and reduced intraoperative bleeding. 1 pt. with epidural and subdural local fistula, 1 pt. with acute intracerebral hemorrhage for tumor regrowth.	unclear
Truckenmueller	Frontiers in oncology	2022	retrospective cohort study	PSMA PET	LuPSMA	6.03 (5.74–6.10) GBq/cycle for 2 cycle every 9–11 wks	correlation between [68 Ga]Ga-PSMA uptake and histological PSMA expression, TBR and rLuPSMA	NA	CTCAE v 5.0	yes	3	1 glioblastoma and 2 astrocytoma	yes	20 pts. included mSUVmax 4.5 (3.7–6.2)High TBR correlated with increased endothelial PSMA expression. Only 3 pts. had TBRmax>1.0 and qualified for LuPSMA RLT. No SE.observed. No efficacy data	maximum 15 weeks
Heute	JNM	2010	prospective	DOTA PET	YDOTA	TCA 1.7–2.2 GBq in 3 or 4 cycles locally injected into a previously implanted catheter system every 3 mo	Efficacy and safety	FUI: ceMRI DOTA PET, FDG PET, FET PET	unknown	yes	3	Grade 4 glioblastoma	yes	Treatment successful in all 3 patients, with only minor SE, such as epileptic seizure, transient, mild headache 1 pt. CR, 2 pts. PR clinical improvement and improved QOL	Unclear, probably 4 years
Cordier Forrer Bruchertseifer	EJNMMI	2009	Pilot trial	^−^	^213^Bi-DOTA -substance P	Intratumoral injection, 3–5 injections over 2 days. 4 pts. with TCA 1.07–2.00 GBq, 1 pt. TCA 7.36 GBq divided into 4 cycles	Feasibility and safety	MRI	CTCAE v2.0	Yes (with blood sampling)	5	glioma (grade 2–4)	yes	feasible and tolerated without additional neurological deficit. No relevant AE. Possible radiation induced necrosis	unclear
Nemati	CNM	2021	prospective	Octreoscan® and DOTA PET	LuDOTA	1–4 cycles every 1–2 mo, TCA 3.7–26.9 GBq	Efficacy, safety, qol	RANO, Karnoski performance status	CTCAE 4.03	yes	16	3 and 4	yes	CR in 2 pts., PR in 5 pts., SD in 3 pts., PD in 6 pts. No relevant SE. No significant improvement of qol	1–26 months

This table provides a comprehensive summary of clinical studies, including in particular imaging modalities, study design, primary objectives, post therapy WBS and/or dosimetric evaluations, patient and tumor characteristics and outcomes in terms of efficacy and safety. Both prospective and retrospective studies, as well as pilot and pivotal trials, are included. The table highlights feasibility, tolerability and potential clinical benefit of RLT in malignant gliomas, with generally low rates of severe adverse events.

RP: radiopharmaceutical; FU: follow-up; Mo: months; Octreoscan®: [^111^In]In-DTPA-octreotide scintigraphy; DOTA PET: [^68^ Ga]Ga DOTA PET; LuDOTA: [^177^Lu]Lu DOTA-TATE or [^177^Lu]Lu-DOTA-TOC; YDOTA: [^90^ Y]Y DOTA-TOC; HT: hematological toxicity; NT: neurological toxicity; RN: renal toxicity; DCR: disease control rate; PR: partial responders; SD: stable disease; PD: progression disease; CR: complete responders; TTP: time to progression; CI: clinical improvement; 3DVGR: MRI three-dimensional volume growth rate; TR: PRRT radionuclide tumour retention; [^177^Lu]Lu-DOTA-JR11: LuJR11; SE: side effect; MS: Mean survival; Y: yes; N: no; WBS: Post-therapy whole-body scintigraphy (single or serial studies).

**Table 5:** Published clinical experiences with RLT in meningiomas and gliomas.

First author	Tumor	Study design	RF	Main findings
Minczeles	meningioma	Retrospective	LuDOTA	Efficacy: mOS 13.6 mo
Gerster-Gilliéron.	meningioma	Prospective phase 2	YDOTA	Efficacy: mPSF at least 24 mo.
Minutoli	meningioma	Retrospective	[^111^In]In Pentetreotide	Toxicity: no G3-G4. Efficacy (DCR): PR in 2 pts., SD in 5 patients, PD in 1 PT
Bartolomei	meningioma	Prospectic	YDOTA	Efficacy: SD in 19 and PD in10, median OS 40 months. Median TTP: 61 months in grade 1 and 13 in grade 2 and 3. No NT, no G3 or G4 other toxicity
Kreissl	meningioma	Pilot trial	LuDOTA	Efficacy: SD in 8/10
Hanscheid	meningioma	retrospective	LuDOTA	A single post-therapeutic measurement could estimate the absorbed dose
Vonken	meningioma	retrospective	LuDOTA (high affinity)	Toxicity:1 pt. with G3 HT (Leukopenia). Efficacy: 3 pts. completed treatment: 1 was PR, 2 SD. They all Improved clinical conditions
Amerein	meningioma	retrospective	LuDOTA (high affinity)	HT: 3 pts. with G3 thrombocypenia, 5 with G3 lymphocytopenia and 1 with G4 lymphocytopenia. RN: 1 pt. with G3-G4, 1 pt. with local necrosis. Efficacy: 1/10 CR, 1/10 PR, 8/10 SD, 9/13 CI m PFS of 18 mo
Hartrampf	meningioma	retrospective	LuDOTA	no toxicities; mPFS 13.8–107.7 mo, OS 38.2 to 111.4
Kertels	meningioma	retrospective	LuDOTA	HT: 5 pts. anemia>G3, 7 pts. trombocytopenia >G3, 9 pts. leukopemia >G3. Efficacy: mPSF 12 mo, OS 37 mo. SD in 6 pts.
Kletting	meningioma	retrospective	YDOTA	Optimal activity of 4.2 ± 1.8 GBq for meningioma
van Essen	meningioma	Prospective?	LuDOTA	Efficacy: 4 PD, 1 SD. Toxicity uncleae
Graillon	meningioma	retrospective	LuDOTA	3DVGR significantly decreased at 3, 6, and 12.
Hänscheid	meningioma	Pilot trial	LuDOTA	Strong correlation between TR ansd SUV
Eigler	meningioma	prospective phase 0	LuDOTA	Toxicity: 2 pts. with G3 lymphopenia and 1 pt. with G3 lymphopenia and neutropenia after DOTA-JR11. Efficacy DRC: 83%
Puranik.	meningioma	Retrospective	LuDOTA	Efficacy: median TTP of 8.9 months, PD in 2 pts., PR in 2 pts., 4 pts. in SD Toxicity: unclear
Severi	meningioma	Prospective	LuDOTA and YDOTA	Efficacy: DCR of 57%, mPFS of 16 mo, mOS 36 mo. Toxicity: G3 platelets toxicity in 1 pt. No symptomatic worsening of conditions. Efficacy: For rechallenge: mPFS of 6.5 mo and mOS of 17 mo
Reuvers	meningioma	unknown	LuDOTA	PRRT induced DNA damage, correlated with SSR expression
Dubois	meningioma	restrospective	LuDOTA	Toxicity: 14 pts. with G3 or higher HT with a single parameter;3 pts. with 2 parameters; 4 pts. with 3 parameters; and 2 pts. with 4 parameters. 1 pt. had G3 hepatic cytolysis. Risk factors not considered separately for meningioma
Salgues	meningioma	restrospective	LuDOTA	Toxicity: frequent SE transient G1 HT. 3 pts. G3 lymphocytopenia. Efficacy: 5/6 pts. with SD at 12 months. PFS at 6mo was 85.7% and PFS at 12mo was 66,7%
Seystahal	meningioma	retrospective	LuDOTAand YDOTA	Efficacy: SD in 10/20, mPFS and PFS at 6 mo stratified according to grade, as such as mOS. Ga DOTA uptake was linked to MRI
Marincek	meningioma	Prospective phase 2	LuDOTA and YDOT.	Efficacy: SD in 23 pts. Relevant HT in 3 pts. and severe RT in 1 pt. MS of 8.6 y from recruitment. SD and high tumor uptake associated with longer survival.
Li	glioma	Prospective phase 2	^125^I-mAb425	mOS 15.7 mo, 1 y survival 62.5%, 2 y survival 25.5%, better in RIT.7 (3.6%) pts. with acute SE mostly G1-G2 (flushing, nausea, hypotension, skin irritation at the injection site). 4 patients became HAMA positive No Grade 3 or 4 toxicities. TMZ + RIT groups no major toxicities
Keinfel	glioma	prospective	[⁹⁰Y]Y-DOTA GAsubstance P	Very good agreement between pre- and post-therapeutic dosimetry Good correspondence between the pretherapeutic test injection and the dose deposition
Krolicki	glioma	Pivotal study	[^213^Bi] Bi/^225^[Ac] Ac DOTA-substance P	RFS of 2–16 ys in astrocytoma (8 pts) and of 4–24 ys in oligodendroglioma (3pts) Low neurotoxicity
Cordier-Forrer Kneifel	glioma	Prospective phase 1	[⁹⁰Y]Y-DOTA DOTAGA–substance P	No relevant SE. No increased intracranial pressure. During surgery better resectability and reduced intraoperative bleeding. 1 pt. with epidural and subdural local fistula, 1 pt. with acute intracerebral hemorrhage for tumor regrowth.
Truckenmueller	glioma	retrospective cohort study	LuPSMA	20 pts. included mSUVmax 4.5 (3.7–6.2)High TBR correlated with increased endothelial PSMA expression. Only 3 pts. had TBRmax>1.0 and qualified for LuPSMA RLT. No SE.observed. No efficacy data
Heute	glioma	prospective	YDOTA	Treatment successful in all 3 patients, with only minor SE, such as epileptic seizure, transient, mild headache 1 pt. CR, 2 pts. PR clinical improvement and improved QOL
Cordier Forrer Bruchertseifer	glioma	Pilot trial	^213^Bi-DOTA -substance P	feasible and tolerated without additional neurological deficit. No relevant AE. Possible radiation induced necrosis
Nemati	glioma	prospective	LuDOTA	CR in 2 pts., PR in 5 pts., SD in 3 pts., PD in 6 pts. No relevant SE. No significant improvement of qol

This table summarizes key results of the comparison between published studies on meningiomas and gliomas. Study design, radiopharmaceuticals and main findings were considered. Overall, these studies demonstrate feasibility, tolerability and potential clinical benefit of RLTs in both tumor types. Notably, meningioma studies predominantly used 177Lu- and 90Y-labeled DOTA-peptides, whereas glioma studies included RIT and alpha targeted therapies. Glioma studies were generally smaller and more heterogeneous.

RP: radiopharmaceutical; FU: follow-up; Mo: months; Octreoscan®: [^111^In]In-DTPA-octreotide scintigraphy; DOTA PET: [^68^ Ga]Ga DOTA PET; LuDOTA: [^177^Lu]Lu DOTA-TATE or [^177^Lu]Lu-DOTA-TOC; YDOTA: [^90^ Y]Y DOTA-TOC; HT: hematological toxicity; NT: neurological toxicity; RN: renal toxicity; DCR: disease control rate; PR: partial responders; SD: stable disease; PD: progression disease; CR: complete responders; TTP: time to progression; CI: clinical improvement; 3DVGR: MRI three-dimensional volume growth rate; TR: PRRT radionuclide tumour retention; [^177^Lu]Lu-DOTA-JR11: LuJR11; SE: side effect; MS: Mean survival; Y: yes; N: no; WBS: Post-therapy whole-body scintigraphy (single or serial studies).

#### Studies about meningiomas

##### Patients selection according to SSRs

In 12 studies patients were selected according to DOTA PET; in six studies, selection was based on Octreoscan®; in two studies, both modalities were used; and in two studies the selection method was not reported. In particular, Octreoscan® alone was used in studies published between 2006 and 2017, whereas DOTA PET alone was used in studies published between 2012 and 2024.

#### Study design

13 Studies were retrospective (59.1%), six were prospective (27.3%), two (9.1%) were pilot trials, in one case (4.5%) the study could not be classified in this sense.

#### Radiopharmaceutical and treatment schedule

In 15 studies (68.3%), patients were treated with LuPRRT; in three studies (13.6%), with YPRRT; in another three studies (13.6%), with a combination of Lu- and Y-based PRRT; and in one study (4.5%), with [^111^ In]In-Pentetreotide, administered in two patients together with a beta-emitting radiolabelled peptide (13). In the case of LuPRRT, total cumulative activity (TCA) amounted between 7.4 (14) and 39.5 GBq (15). With the exception of two studies, where intra-arterial usage was required, the administration method was intravenous. In the case of intra-arterial administration, [^177^Lu]Lu- high-affinity (HA)-DOTATATE was used: It exhibits some structural and functional differences when compared to the DOTATATE compound currently employed (16, 17). In the phase-0 PROMENADE study, the use of LuDOTA was combined with the use of [^177^Lu]Lu-DOTA-JR11 (18). This latter agent is a radiolabelled SSRs antagonist designed for PRRT. Unlike somatostatin receptor agonists, which internalize upon binding to the receptor, JR11 binds to a broader range of SSR subtype 2 sites. This includes both internalizing and non-internalizing sites, leading to higher tumor uptake and prolonged tumor retention.

In the case of YPRRT, the TCA ranged between 7.4 GBq (19) and 15 GBq (20). In one prospective phase 2 study, which combined the use of LuPRRT and YPRRT, the cycles were repeated until progression or until toxicity manifests (21).

#### Main topic and evaluation criteria

Safety was the main topic in 17 studies (77.3%) and the registration of the adverse events (AEs) was its endpoint (20, 22).

Efficacy was evaluated in 16 studies (72.7%), with the endpoints of progression-free survival (PFS) (2, 15, 17, 19, 23, 24), disease control rate (DCR) (2, 13, 20, 25), OS (15, 23), and time to progression (TPP) (20, 25). Response criteria were variable: In eight studies, they were based on Response Assessment in Neuro-Oncology Working Group (RANO) criteria (26); in three on Southwest Oncology Group standard response criteria (SWOG) criteria (27); and in two on Response Evaluation Criteria in Solid Tumors (RECIST) criteria (28).

#### WBS

WBS was performed in 16 studies (72.7%), mostly using LuPRRT. In four cases, OLINDA/EXM software was used. Dosimetry was the main topic in two studies (18, 25, 29, 30). Only two of these studies reported the use of YPRRT. In particular, one study aimed exclusively to optimize the peptide amount and activity for YPRRT, while in the other, a sub-cohort of 14 patients received co-infusion of [^111^In]In DOTA^−^TOC to assess YPRRT biodistribution (20).

#### Main findings

With regard to safety, the authors primarily focused on major toxicities, of hematological or renal nature, as shown in the synoptic table. In a study by Severi, patients were treated with YPRRT (five) or LuPRRT (37) and with different TCA, based on the presence of risk factors for toxicity. According to this study, the treatment was well tolerated, and only one patient had G3 toxicity on platelets. No deterioration of clinical conditions occurred in any case. Some authors reported no major (G3 or G4) toxicities with YPRRT (20), LuPRRT (23) or with [^111^In]In Pentetreotide (13).

In other studies, toxicities were more significant. Amerein reported retrospective data on patients treated with intra-arterial HA-LuPRRT. Three patients experienced G3 thrombocytopenia, five had G3 lymphocytopenia, and one developed G4 lymphocytopenia. In addition, one patient showed transient G3/G4 renal toxicity, probably unrelated to PRRT, and another developed local necrosis likely associated with angiography. Kertels also reported higher toxicity rates in patients with meningioma and neurofibromatosis treated with LuPRRT, describing severe anemia in five cases, severe thrombocytopenia in seven and severe leukopenia in nine.

Eigler and the PROMENADE group conducted a phase-0 study on patients treated with one cycle of LuPRRT followed by two or three cycles of [177Lu]Lu-DOTA-JR11 PRRT. They reported G3 lymphopenia in two patients, and combined G3 lymphopenia and neutropenia in one patient.

Finally, in a study by Dubois, short-term toxicity prediction was retrospectively investigated in patients with SSRs tumors treated with LuPRRT. Only five of them were affected by meningioma. The authors reported considerations about the increased risk of toxicity generally, without separating between the different primary tumours, and found that risk factors were gastrointestinal primary tumor diagnosis, bone metastases, peritoneal metastases, pancreatic metastases or pulmonary metastases, and high tumor grade (31).

With regard to efficacy, DCR was investigated by Severi and it was reported of 57%. Other studies on LuPRRT reported stable disease (SD), respectively, in 8/10 patients (14, 17), in 4/5 patients (22), and in 5/6 patients (32). In patients treated with [^111^In]In Pentetreotide SD was found in 5/8 patients[13]. When associated with antagonist, according to the study on [^177^Lu]Lu-DOTA-JR11 PRRT reported above, the DCR could reach 83% (18). Together with DCR, also PFS and OS were considered. In the study by Hartrampf, patients were treated with the combination of LuPRRT and EBRT with a long-term follow-up (median of 105 months). Median PFS (mPFS) was reported ranging from 13.8 to 107.7 months, median OS (mOS) from 38.2 to 111.4 months.

To provide an alternative definition of treatment response, some researchers looked for a volumetric parameter. A study by Graillon evaluated the impact of PRRT with Lutathera in nonanaplastic meningiomas, according to the three-dimensional volume growth rate (3DVGR) measured with MRI (33). 3DVGR significantly decreased at 3, 6, and 12 months after treatment initiation, analysing each lesion separately. In particular, at 3, 6, and 12 months after treatment initiation, 4/8, 6/7, and 5/6 patients were class 2 (stabilization or severe 3DVGR slowdown), respectively. Moreover, its antitumor activity persisted for 12–18 months following treatment initiation (34).

Other studies focused on dosimetry. Hänscheid retrospectively assessed activity kinetics from planar images in patients with various tumors, including meningioma, treated with LuPRRT. Mono- or biexponential functions were applied to data from the kidneys, liver, spleen, and neuroendocrine tumor (NET) lesions. Tissue-specific deviations of the approximation from the time integral were calculated at 24, 48, 72, 96, 120, and 144 h. The authors concluded that a single quantitative measurement of abdominal activity concentration by SPECT/CT, performed four days after PRRT, could provide a three-dimensional dose map and estimate the actual absorbed doses.

In a study by Kletting, a whole-body physiologically based pharmacokinetic (PBPK) model was developed to determine in YPRRT biologically effective doses (BEDs) to the tumor, to the liver, to the spleen, and to the red marrow for a maximal kidney BED (20 Gy _2.5_) for different peptide amounts and activities. The authors found that these two parameters depended on tumor perfusion and receptor density. For meningioma and for YPRRT, the optimal amount and pertaining activity was found to be 76 ± 46 nmol (118 ± 71 μg) and 4.2 ± 1.8 GBq.

Finally, to assess the short-term response to radiation therapies such as PRRT and EBRT, Reuvers et al. developed a 3D spheroidal culture model for meningiomas. The authors noticed that in general meningioma spheroids retained characteristics of the parental tumor during the initial days of culturing, but a subset of tumors veered toward a more aggressive phenotype. PRRT induced DNA damage which was detectable for an extended timeframe as compared to EBRT. Furthermore, levels of DNA damage in spheroids after PRRT correlated with SSR2-expression levels of parental tumors (35).

#### Follow-up times

Follow-up times were extremely variable, ranging from 3 months (36, 37) to a median of 105.0 months (23).

#### Studies about gliomas

##### Patients’ selection according to SSRs

Given the heterogeneity of the radiopharmaceuticals used, only in two cases patients were selected according to SSRs imaging and in particular with DOTA PET (38) and both (DOTAPET and Octreoscan®) (39). In one study, patients were selected through [^68^Ga]Ga-PSMA (PSMA PET) (40).

#### Study design

Three of the included studies were prospective (39, 41, 42); one was a retrospective cohort study (40); one was a pilot trial (43); and for the other three studies, a similar classification was not possible (38, 44, 45).

#### Radiopharmaceutical and treatment schedule

YPRRT was proposed in the study by Heute on three patients. A TCA of 1.7–2.2 GBq was delivered in three or four cycles and locally injected into a previously implanted catheter system every 3 months (38).

LuPRRT was proposed in a study by Nemati, who treated 16 patients for 1–4 cycles every 1–2 months and with a TCA of 3.7–26.9 GBq.

Other radiopharmaceutical used were:

- [^125^I]I-labeled anti-epidermal growth factor receptor 425 murine monoclonal antibody (^125^I-mAb425) is a murine monoclonal antibody directed against the epidermal growth factor receptor (EGFR) and conjugated with ^125^I. This antibody binds to a part of the extracellular domain of human EGFR. The used schedule was of 1.8 GBq over a course of three weekly administration and for a TCA of 5.4 GBq, approximately 4–6 weeks after surgery and EBRT. A total of 192 patients were treated over a course of 20 years, starting from 1987 (41).
- [^111^ In]In/[^90^Y]Y –DOTAGA substance P, used in two studies before surgery and through intratumoral injections. Substance P is the main ligand of neurokinin type 1 (NK-1) receptors, commonly overexpressed in gliomas and tumor neovasculature. One of the two studies was addressed to dosimetry and, in particular, to establish a protocol for intratumoral radiopeptide using 2 MBq of [^111^ In]In-substance P and 370–3.330 MBq of /[^90^Y]Y-substance P (44). The other study was addressed to evaluate feasibility of intratumoral injection of [^90^Y]Y-DOTAGA–substance P at weekly intervals, with a TCA of 120–345 mCi with dose escalation and before surgery (42).
- [^213^Bi] Bi/ [^225^Ac] Ac DOTA-substance P, considered in two studied and based on the combination of targeted alpha therapy (TAT) with a linear, small-peptide vector. [^213^Bi] Bi emits high LET alpha particles (~5–9 MeV, ~100 keV/μm) with a short tissue range (< 0.1 mm), ^225^Ac has longer half-life (~9.9 days), decays via a cascade yielding ^{213}Bi among daughters, delivering up to five alpha particles per decay. The alpha-particle radiopharmaceuticals are considered to delivery radiation to malignant sites while sparing healthy tissue. In one study, addressed to efficacy and toxicity in 11 patients, 2–2.5 GBq of [^213^Bi]Bi DOTA-substance P or 17–35 MBq [^225^Ac]Ac DOTA-substance P were locally injected directly into the tumor via a stereotactic insertion of a capsule-catheter system (45). In another study, the radiopharmaceutical was used in five patients through intratumoral injection, three to five times and over 2 days, with a TCA of 1.07–2.00 GBq in 4 cases and of 7.36 GBq divided into 4 cycles in one case (43).

LuPSMA was considered in one study (40). Several previous studies had shown that PSMA is overexpressed in the tumor-associated neovasculature of high-grade gliomas (46–48), so building on this premise, 20 patients were firstly evaluated with [^68^Ga]Ga-PSMA PET/MRI (PSMA PET). Three of them likely to benefit from LuPSMA according to the maximum tumor-to-background ratio (TBRmax) of tumor and liver, which had been chosen by the authors as a criterion for recommending LuPSMA, with a predefined cut-off set at 1. In 11 samples, immunohistochemical PSMA expression was determined using the H-score (49) and correlated with uptake on PSMA PET. The H-score in the three patients eligible for LuPSMA was higher than the H-score in patients who were not (respectively 65 versus 30, *p* = 0.08). While this review primarily focuses on primary brain tumors, it is essential to recognize that LuPSMA may play a role also in brain metastases, according to recent evidence from the literature (50).

#### Main topics and evaluation criteria

In seven studies, safety was the main topic and the registration of the adverse events (AEs) its endpoint (38, 41–43, 45). Four studies regarded efficacy, in terms of recurrence-free survival and DCR (38, 39, 45).

One study regarded dosimetry (44). It was conducted through injections of [^111^ In] In-substance P and [^90^ Y] Y-substance P in 12 patients with malignant gliomas. Over a period of 24 h, serial SPECT scans were performed on a dual-head SPECT camera. Quantitative voxelwise dose distribution maps (in Gy/ GBq) were computed from these data and pre- and post-therapeutic values were compared.

#### Post-therapy whole-body scintigraphy (WBS, single, or serial studies)

WBS was performed in all studies, except for two (41, 45).

#### Main findings

With regard to toxicity, in the case of radioimmunotherapy (RIT), the combination with temozolomide (TMZ) was not associated with an increase in toxicity. Adverse events occurred in seven patients (3.6%), and were generally mild, including flushing, nausea, hypotension, and local skin irritation at the injection site. Only four patients developed human anti-mouse antibodies (HAMA). Regarding the studies on TAT, neurotoxicity appeared to be minimal. In the case of [^111^ In]In/[^90^Y]Y –DOTAGA substance P, no short-term side effects were observed, too; moreover, intraoperative bleeding appeared to be reduced. For YPRRT, side effects were mild and an overall improvement in quality of life was reported (38, 41, 42).

In terms of efficacy, the combination of TAT and substance P provided a recurrence-free survival time ranging from 2 to 16 years. This was observed in eight patients with astrocytoma, who were treated with TAT following a biopsy or tumor debulking. Regarding oligodendrogliomas, the recurrence-free survival time was 24 years in the first case treated and 4 and 5 years in the two second-line cases, with low toxicities (45). Furthermore, the use of YPRRT in three patients showed the following results: One patient had a complete response (CR) and two patients a partial response (PR) at follow-up imaging with ceMRI, DOTA PET, [18F]F FDG PET, and [18F]F Fuoroethyltyrosine PET. Clinical improvements and a global improvement in quality of life (qol) was noticed, too (38). Finally, in the study by Nemati on LuPSMA, the radiological response according to RANO criteria was considered: Two patients showed a CR, five patients a PR, three patients a SD, and six patients a progression disease (PD). No significant improvement of qol was noticed.

With regard to the dosimetric study, it was found a very good agreement between pre- and post-therapeutic dosimetry and a good correspondence between the pretherapeutic test injection and the dose deposition (44).

#### Follow-up times

In most cases, the follow-up period was not clearly defined. In one study, it ranged from 1 to 24 years (median 10 years) (45); in another, patients were monitored for up to 15 weeks (40); and in a third study, the follow-up period ranged from 1 to 26 months (39).

#### CASP analysis of the selected studies

**Figure 3:**
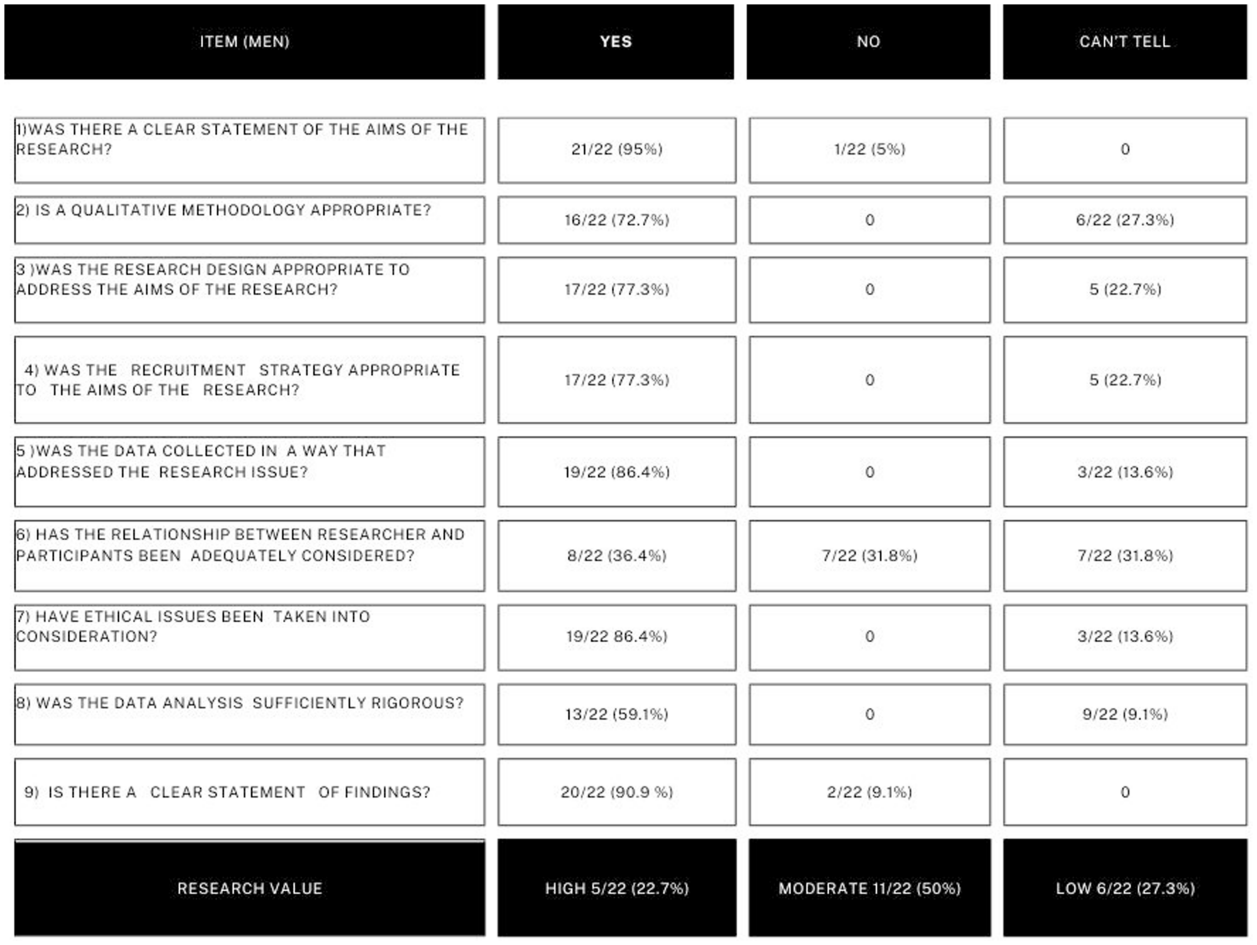
In this figure CASP analysis on meningiomas studies is reported sinoptically.

**Figure 4:**
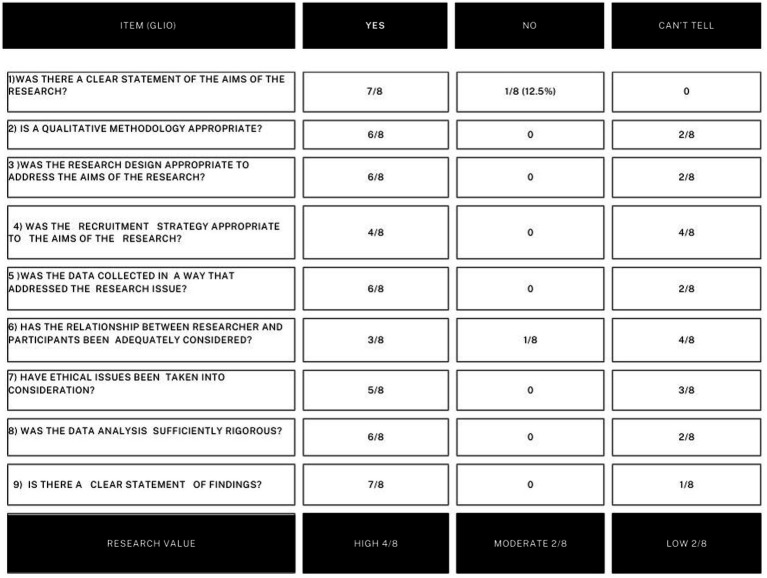
In this figure CASP analysis on gliomas studies is reported sinoptically.

The results of CASP analysis is reported in detail in Table 6 and Figure 3 for meningiomas and in Table 7 and Figure 4 for gliomas. Below, the most noteworthy data are summarized.

**Table 6:** CASP analysis of published RLT studies on meningiomas.

First author	(1) Was there a clear statement of the aims of the research?	(2) Is a qualitative methodology appropriate?	(3) Was the research design appropriate to address the aims of the research?	(4) Was the recruitment strategy appropriate to the aims of the research?	(5) Was the data collected in a way that addressed the research issue?	(6) Has the relationship between researcher and participants been adequately considered?	(7) Have ethical issues been taken into consideration?	(8) Was the data analysis sufficiently rigorous?	(9) Is there a clear statement of findings?	(10) How valuable is the research?
Minczeles	yes	yes	yes	yes	yes	Cannot Tell	yes	Yes	Yes	Low(number of pts)
Gilliéron	yes	Yes	Yes	Yes	Yes	Cannot tell	Yes	Cannot tell (pochi pz)	yes	Moderate
Minutoli	no	Cannot tell	Cannot tell	Cannot tell	Cannot tell	Cannot tell	yes	Cannot tell	yes	Moderate
Bartolomei	Yes	Yes	Yes	Yes	Yes	Yes	Yes	Yes	Yes	high
Kreissl	yes	yes	yes	yes	yes	no	Yes	Cannot tell (pochi pz)	no	low
Hanscheid	Yes	Yes	Yes	Cannot tell	Yes	no	Cannot tell	Yes	Yes	moderate
Vonken	Yes	Cannot tell	Cannot tell	Cannot tell	Cannot tell	no	yes	Cannot tell	yes	Low (very small number of pts)
Amerein	yes	yes	yes	yes	yes	yes	yes	yes	yes	Moderate
Hartrampf	Yes	yes	Yes	Yes	Yes	Yes	yes	Yes	Yes	Moderate
Kertels	Yes	yes	Yes	Yes	Yes	Yes	yes	Yes	Yes	High
Kletting	Yes	Yes	Yes	Yes	Yes	Cannot tell	Cannot tell	Yes	Yes	Low (very small number of pts)
van Essen	Yes	Cannot tell	Cannot tell	Cannot tell	Yes	Yes	Yes	Cannot tell	Yes	Low (very small number of pts)
Graillon	Yes	yes	Yes	Yes	Yes	Yes	yes	Yes	Yes, but complexly (in modo complesso)	high
Hänscheid	Yes	Cannot tell	Cannot tell	Cannot tell	yes	no	yes	Cannot tell	yes	moderate
Eigler	Yes	yes	yes	yes	yes	Cannot tell	Yes	Yes	yes	moderate (very small number of pts)
Puranik	Yes	Cannot tell	Cannot tell	Yes	Yes	No	yes	Cannot tell	No	low
Severi	Yes	Yes	Yes	Yes	Yes	Yes	Yes	Yes	Yes	high
Reuvers	yes	Yes	Yes	Yes	Yes	Cannot tell	Cannot tell	Yes	Yes	Moderate/high
Dubois	Yes	Cannot tell	Yes	Yes	Cannot tell	no	Yes	Cannot tell	Yes	moderate
Salgues	Yes	Yes	Yes	Yes	Yes	no	Yes	Cannot tell	Yes	moderate
Seystahal	Yes	Yes	Yes	Yes	Yes	Cannot tell	Yes	Yes	Yes	moderate
Marincek	Yes	Yes	Yes	Yes	Yes	Yes	Yes	Yes	Yes	high

In this table a methodological quality assessment was performed. Following the CASP critical appraisal guidelines, key aspects included: clarity of research aims, appropriateness of study design, recruitment strategy, data collection and analysis, consideration of researcher-participant relationships, ethical compliance and clarity of findings. Studies on meningiomas generally demonstrated consistent methodological approaches.

**Table 7:** CASP analysis of published RLT studies on gliomas.

First author	(1) Was there a clear statement of the aims of the research?	(2) Is a qualitative methodology appropriate?	(3) Was the research design appropriate to address the aims of the research?	(4) Was the recruitment strategy appropriate to the aims of the research?	(5) Was the data collected in a way that addressed the research issue?	(6) Has the relationship between researcher and participants been adequately considered?	(7) Have ethical issues been taken into consideration?	(8) Was the data analysis sufficiently rigorous?	(9) Is there a clear statement of findings?	(10) How valuable is the research?
Li	Yes	Yes	Yes	Cannot tell	Yes	Yes	Cannot tell	Yes	Yes	High
Keinfel	Yes	Yes	Yes	Yes	Yes	Cannot tell	Yes	Yes	Yes	High
Krolicki	no	Cannot tell	Cannot tell	Cannot tell	Cannot tell	no	Cannot tell	Cannot tell	Cannot tell	low
Cordier- Forrer-Kneifel	Yes	Yes	Yes	Yes	Yes	Cannot tell	Yes	Yes	Yes	high
Truckenmueller	Yes	Yes	yes	Cannot tell	yes	Cannot tell	yes	yes	yes	low
Heute	yes	Cannot tell	Cannot tell	Cannot tell	Cannot tell	Cannot tell	Cannot tell	Cannot tell	yes	moderate
Cordier Forrer Bruchertseifer.	Yes	Yes	Yes	Yes	Yes	Yes	Yes	Yes	Yes	high
Nemati	Yes	Yes	Yes	Yes	Yes	Yes	Yes	Yes	Yes	moderate

In this table, a methodological quality assessment was conducted. As for published studies on meningiomas, the CASP critical appraisal guideline were applied. Key aspects regarded mostly: study rigor, data collection and analysis and ethical compliance. Overall, most studies demonstrated rigorous design and reporting. Some studies showed limitations in qualitative methodology or reporting completeness.

Regarding meningiomas, with the exception of one paper, all of the works had clear aims. The statement of findings was evidently reported in 20/22 (91%) of the papers, too. The general quality of the researches and the quality of the methodology, of the study design, of the recruiting strategy, and of the data analysis were variable. In more detail, the quality of the methodology was considered appropriate in 16/22 (72.7%) of the studies, and the study design was considered adequate in 17/22 (77.3%), as was the recruiting strategy. The overall value of the research was found high in 5/22 (22.7%) of the papers, moderate in 11/22 (50%) of them, and low in 6/22 (27.3%).

Regarding gliomas, all of the works had clear aims but one, as well as regards the clear statements of findings.

The following parameters, however, showed variability: The ethical issues, which have been taken into consideration in five out of eight studies, the quality of the methodology, which was considered as appropriate in six, the quality of the research design, considered as appropriate in six, the quality of the recruiting strategy, considered as adequate in four.

The general quality of the research resulted high in four studies, moderate in two, and low in two.

### Clinical trials

#### Search strategy

**Figure 5:**
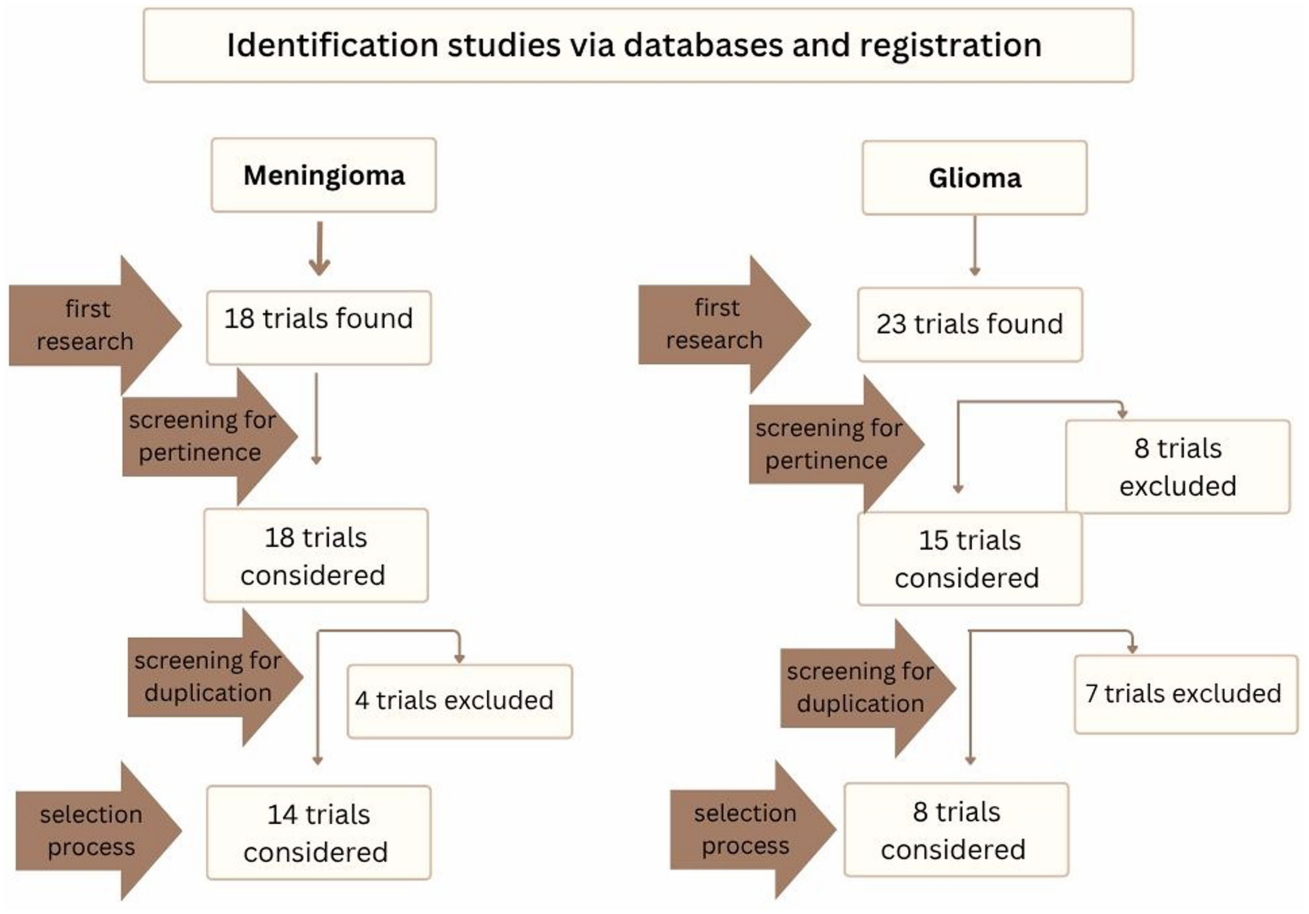
In this figure the selection process for clinical trials is reported, from the first research to selection process.

The results of the search strategy is reported in Figure 5.

Fourteen studies on meningiomas and eight on gliomas were included in the final analysis. The characteristics of the trials on meningiomas and gliomas are presented in detail in Tables 8, 9, respectively, so what follows is strictly an overview of the trials.

**Table 8:** Overview of clinical trials of RLT in meningiomas.

Title	Study site (s)	First submitted (year)	Radiopharmaceutical	Study type	Main endpoints	Status
(1) Dosimetry Guided PRRT with 90Y-DOTATOC	University of Iowa	2017	Y DOTA	Phase 2	safety and efficacy of 90Y-DOTATOC, the role of 68Ga-DOTATOC	completed
(2) Treatment of Recurrent or Progressive Meningiomas With the Radiolabelled Somatostatin Antagonist 177Lu-satoreotide	University Hospital, Basel, Switzerland	2021	[^177^Lu]Lu Satoreotide	phase 0 study followed by Phase I/II study.	Comparison of the therapeutic index of 177Lu-DOTA-JR11 and 177Lu-DOTATOC, dosimetry (tumour absorbed dose), early onset toxicity, QOL	recruting
(3) Peptide Receptor Radionuclide Therapy Administered to Participants With Meningioma With 67Cu-SARTATE	Royal North Shore Hospital Sydney	2019	[^67^ Cu]Cu-SARTATE™	Phase 1/2	Safety and tolerability of multiple doses of Cu-67 SARTATE, dosimtery	completed
(4) Semiautomated Segmentation Methods of SSTR PET for Dosimetry Prediction	Central Hospital, Nancy, France	2022	LuDOTA	Observational retrospective	Correlation between SUVmean and SUVmax for tumor absorbed dose, a new semi-automated segmentation for determining pretherapy metabolic tumor volume	completed
(5) Combination of Everolimus and 177Lu-DOTATATE in the Treatment of Grades 2 and 3 Refractory Meningioma: a Phase IIb Clinical Trial	Central Hospital, Nancy, France	2023	LuDOTA and Everolimus	Phase 2	PFS, OS, QOL, toxicity	recruting
(6) Radiolabeled Octreotide in Treating Children With Advanced or Refractory Solid Tumors	University of Iowa	2002	YDOTA	Phase 1	safety and effectiveness of radiolabeled octreotide in treating children	completed
(7) Lutathera for the Treatment of Inoperable, Progressive Meningioma After External Beam Radiation Therapy	Mayo Clinic	2020	LuDOTA	Phase 2	Efficacy, safety, PFS, OS, QOL, dosimetry	recruting
(8) 177Lu-DOTATATE for Recurrent Meningioma (LUMEN-1)	France, Norway, Austria	2024	Local standard of Care vs. LuDOTA	Phase 2, Randomized	PFS, OS, Radiological response rate, QOL	recruiting
(9) Semi-automatic Segmentation Method for Determining 177Lu-DOTATATE Tumor Dosimetry completato	Central Hospital, Nancy, France	2024	LuDOTA	Observational Cohort Time Perspective Retrospective	Dice index between semi-automatic segmentation and reference segmentation	completed
(10) Phase II Study of 177Lu-DOTATATE Radionuclide in Adults With Progressive or High-risk Meningioma	NYU Langone Health	2019	LuDOTA	Phase 2	PFS, ORR, OS	active not rectruinting
(11) MOMENTUM-1: A Multicenter, Randomized, Open-Label, Phase II Study of [177 LU]LU-DOTATATE in Adults With Progressive Intracranial Grade 1–3 Meningioma	RTOG Foundation, Inc	2025	LuDOTA	Phase 2, randomized	Comparing [177Lu]Lu-DOTATATE to Standard of Care in tems of PFS, OS, DCR, toxicity	not yet recritug
(12) Tumor Absorbed Dose–Response Relationship in Patients Treated With 177Lu-DOTATATE for Meningioma (DATUM)	Central Hospital, Nancy, France	2024	LuDOTA	Observational Cohort Retrospective	diagnostic accuracy for PFS	completed
(13) Lutathera for Treatment of Recurrent or Progressive High-Grade CNS Tumors	Nationwide Children’s Hospital		LuDOTA	Phase 1/2	MTD, recommended Phase II dose (RP2D) toxicity, PFS	recruting
(14) Study in Children and Adolescents of 177Lu-DOTATATE (Lutathera®) Combined with the PARP Inhibitor Olaparib for the Treatment of Recurrent or Relapsed Solid Tumours Expressing Somatostatin Receptor (SSTR) (LuPARPed). (LUPARPED)	Fundación de investigación HM		LuDOTA (+ OLAPARIB)	Phase 2	ORR	recruting

This table summarizes study design, the proposed radiopharmaceuticals, primary endpoints and study status of the trials. Early-phase and randomized trials are reported. Regarding radiopharmaceuticals, they are also combined with targeted agents such as everolimus or olaparib. Most studies focus on safety, dosimetry, efficacy and quality of life. Overall, these trials demonstrate the feasibility and translational potential of RLT in meningiomas.

LuDOTA: [^177^Lu]Lu DOTA-TATE or [^177^Lu]Lu-DOTA-TOC or LUTATHERA ®; YDOTA: [^90^ Y]Y DOTA-TOC; QOL: Quality Of Life; ORR: overall response rate (ORR); MTD: maximally tolerated dose.

**Table 9:** Overview of clinical trials of RLT in gliomas.

Title	Study site (s)	First submitted (year)	Radiopharmaceutical	Study type	Main endpoints	status
1. Yttrium Y 90 SMT 487 in Treating Patients With Refractory or Recurrent Cancer	? in USA	2000	[^90^Y]Y-SMT 487	Phase 1	MTD and safety	completed
2. A Feasibility Study to Evaluate the Safety of the TheraSphere Glioblastoma (GBM) Device in Patients With Recurrent GBM	California, Florida, Illinois, Maryland USA	2022	Y-90 Glass Microsphere System (TheraSphere GBM)	Phase 1	PFS, OS, ORR, toxicity	recruting
3. 68Ga/177Lu-PSMA Theranostics in Recurrent Grade 3 and Grade 4 Glioma	St. Olavs Hospital Norway	2022	[^177^Lu]Lu-PSMA I&T	pilot-study	Efficacy, toxicity, dosimetry, QOL; role of [^68^ Ga]Ga-PSMA uptake	recruting
4. 177Lu-J591 Antibody in Patients With Nonprostate Metastatic Solid Tumors	Weill Medical College of Cornell University, New Yors, USA	2005	[^177^Lu]Lu Radiolabeled Monoclonal Antibody HuJ591	Phase 1	DCR, safety	completed
5. Dose Finding Study of [177Lu]Lu-NeoB in Newly Diagnosed Glioblastoma and in Recurrent Glioblastoma	Novartis Pharmaceuticals	2023	[^177^Lu]Lu -NeoB in combination with RT and TMZ	Phase 1	DLTs toxicity, PFS, OS,	recruiting
6. Radioimmunotherapy with Lu-177 Labeled 6A10 Fab-fragments in Patients with Glioblastoma After Standard Treatmen	University Hospital Muenster, Germany	2022	-[^177^Lu]Lu - Labeled 6A10 Fab-fragments	Phase 1	Pharmacokinetics, absorbed dose, PFS and OS	recruiting
7. A Dose Finding Study of [177Lu]Lu-DOTA-TATE in Newly Diagnosed Glioblastoma in Combination With Standard of Care and in Recurrent Glioblastoma as a Single Agent.	Novartis Pharmaceuticals	2021	[^177^Lu]Lu-DOTA-TATE	Phase Ib	DLTs, PFS, OS, ORR, dosimetry	recruiting
8. A Phase I Study of [177Lu]Lu-FF58 in Patients With Advanced Solid Tumors.	Novartis Pharmaceuticals	2023	[^177^Lu]Lu-FF58	Phase 1	Dose escalation and dose expansion	Terminated

This table summarizes study design, the proposed radiopharmaceuticals, primary endpoints and study status of the trials. The trials were early-phase and pilot studies and investigate a range of radiopharmaceuticals. Most studies focus on safety, dose-finding, pharmacokinetics, dosimetry, efficacy and quality of life. Overall, these trials highlight the feasibility, tolerability and translational potential of RLT in gliomas.

MTD: maximum tolerated dose; PFS: Progression-Free Survival; OS: Overall Survival; ORR: Objective Response Rate; QOL: Quality Of Life; DCR: Disease Control Rate; Dose Limiting Toxicity (DLTs).

#### Overview of the selected studies

##### Trials about meningiomas

A total of 10 trials focus on the use of LuPRRT, two on YPRRT, one on the SSRs antagonist Satoreotide labeled with [^177^ Lu] one on [^67^Cu]Cu-SARTATE™. Satoreotide has been tested above all in NETs (51, 52). [^67^Cu]Cu-SARTATE™ consists of SARTATE, an octreotate-derived ligand, radiolabeled with ^67^Cu, a beta-emitting isotope that also provides gamma emissions suitable for imaging, thereby enabling theranostic applications. Tumor types targeted by [^67^Cu]Cu-SARTATE™ potentially include also gastroenteropancreatic GEP-NETs, bronchial NETs and neuroblastomas (53, 54). The association of LuPRRT with other antitumor agents, such as everolimus, an immunosuppressive agent belonging to the class of mechanistic target of rapamycin kinase (mTOR) inhibitors, and olaparib, a poly ADP-ribose polymerase (PARP) inhibitor, was proposed.

Furthermore, eleven trials are prospective and one is retrospective. The studies status is distributed as follows: Six trials are completed, six are recruiting, and two are active but not recruiting.

##### Trials about gliomas

One trial will investigate the use of LuPRRT, one trial of LuPSMA.

The remaining six trials will test, respectively:

- [^90^Y]Y spheres (Glass Microsphere System, TheraSphere GBM), a form of selective internal radiation therapy (SIRT) involving glass microspheres embedded ^90^Y and originally developed for the treatment of unresectable hepatocellular carcinoma (HCC) via intra-arterial delivery (55). In the context of GBM, TheraSphere are administered via intra-arterial cerebral infusion, enabling localized high-dose beta radiation directly to the tumor bed, while minimizing exposure to surrounding healthy brain tissue. This approach aims to overcome the limitations of EBRT and the blood–brain barrier, offering a promising avenue for treating recurrent or resistant GBM.
- [^90^Y]Y-SMT 487, a radiopharmaceutical consisting of the somatostatin analog SMT 487 labeled with ^90^Y. It primarily targets tumors that overexpress SSRs, especially type 2 and has been evaluated above all in NETs (56).
- [^177^Lu]Lu Radiolabeled Monoclonal Antibody HuJ591, a radiolabeled monoclonal antibody that targets PSMA radiolabelled with ^177^ Lu (57).
- [^177^Lu]Lu - Labeled 6A10 Fab-fragments, which consists of Fab fragments derived from the monoclonal antibody 6A10, radiolabeled with ^177^ Lu. The 6A10 antibody targets carbonic anhydrase XII (CA XII), a membrane-associated enzyme overexpressed in several solid tumors, including renal and lung cancers. It is used for its improved tumor penetration and reduced off-target toxicity due to the smaller size of the Fab fragment (58).
- NeoB, a gastrin-releasing peptide receptor (GRPR)-binding peptide, also known as bombesin receptor subtype 2. When labeled with ^177^ Lu, it results in the formation of the radiopharmaceutical [^177^Lu]Lu-NeoB. As a GRPR antagonist, [^177^Lu]Lu-NeoB enables selective radiation delivery to GRPR-expressing tumors with enhanced tumor uptake and faster clearance from non-target tissues compared with agonist radioligands. (59, 60).
- [^177^Lu]Lu-FF58, a radiolabeled peptide designed to target proteins known as integrins: alpha-v beta-3 integrin (αvβ3) and alpha-v beta-5 integrin (αvβ5).

All the trials were prospective. Five are still recruiting while three have completed the enrolment.

## Discussion

There is a critical unmet need for innovative therapeutic strategies to complement the current standard of care in meningiomas and gliomas, particularly in cases where conventional treatments are no longer viable or tolerated.

In this context, RLT holds significant promise due to its theranostic paradigm and personalized approach. More in detail, the theranostic paradigm —“treating what is seen and seeing what is treated”—allows pretherapeutic assessment of receptor expression (e.g., SSRs via DOTA PET or Octreoscan® or PSMA PET tracers) and the personalized approach enables optimized patient selection and dosimetric planning.

Alternative radiopharmaceuticals, including alpha-emitters, may be considered even in patients with low receptor expression, offering reduced risk of edema and collateral damage. RLT can be administered either systemically or locally (e.g., intra-arterial). This approach helps overcome the blood–brain barrier, deliver higher tumor doses, and minimize generalized toxicity. Overall, it is generally well tolerated and has demonstrated potential for disease control in multiple studies.

Following these considerations, which are applicable across studies involving both meningiomas and gliomas, the discussion then diverges to emphasize the contrasting findings reported in the literature concerning the two tumor types.

Published papers on RLT in meningiomas appear more robust and “traditional” and focused predominantly on PRRT, which has demonstrated an acceptable safety profile and encouraging signs of efficacy. Unfortunately, currently approved radiopharmaceuticals are not indicated for the treatment of meningioma, so the available data remain limited, heterogeneous and based on experimental studies. Toxicity is generally minimal and manageable, with hematologic and renal adverse events occasionally reported. It is important to highlight that in the largest study identified (Severi et al.), the treatment course was carefully individualized, particularly with regard to pre-treatment toxicity risk stratification at the time of patient enrollment. Therapy individualization is a key factor also behind the dosimetric studies, whose aim was to optimise PRRT. Toxicity may also be influenced by factors unrelated to the patient, as well as the administration route and the radioligand type. For example, intra-arterial LuPRRT, was associated with high grade hematologic toxicity and one instance of transient high grade renal toxicity, as reported by Amerein. Similarly, Eigler and the PROMENADE group documented relevant hematological toxicities in patients treated with LuPRRT and Lu-DOTA-JR11.

Regarding efficacy, the DCR varied considerably across studies but remained generally encouraging. Evidence also points to potential synergistic benefits when PRRT is combined with EBRT, suggesting opportunities for multimodal strategies, as explored by Hartrampf. In addition to clinical and imaging data, preclinical insights were provided to evaluate meningioma response to PRRT and EBRT, as the 3D spheroid culture model by Reuvers, demonstrating a potential synergy between the two therapies.

CASP analyses confirm methodological rigor. Most reviewed studies clearly defined their research questions, with appropriate study designs and data collection methods.

Clinical trials also tend to focus primarily on already known compounds, with the exception of [^177^Lu]Lu Satoreotide, of [^67^ Cu]Cu-SARTATE™. Finally, they evaluate the association of LuPRRT with everolimus and with olaparib, thereby suggesting the potential for combining PRRT with antitumor agents.

In gliomas, the landscape remains largely experimental and heterogeneous. Published papers show a more pioneering character, evident even upon preliminary examination, particularly in the study design. Notably, three studies reported data from early-phase trials, indicating that the clinical translation of preclinical data remains at an early stage. The radiopharmaceuticals used were newly tested in these clinical settings, such as RIT and alpha emitters. In the study focusing on RIT, the lack of consistent toxicity and encouraging data on efficacy were reported, but the most interesting aspects of the research concern the notable number of patients enrolled and the fact that recruitment spanned over 20 years. Regarding alpha emitters, treatments with ^213^ Bi and ^225^ Ac were proposed, through intratumoral injections. The advantages of this approach are numerous: First of all, a single injection of this radiopharmaceutical was found to be sufficient to achieve long-lasting tumor control, even if closely spaced injections can be also proposed. Furthermore, the local administration and above all the very favorable dose range of alpha emitters have shown evidence of limiting local neurotoxicity and of minimizing the risk of other side effects. Even with the inherent limitation of a small sample size, the results exposed by Krolicki are striking, showing RFS of up to 16 years in astrocytomas and 24 years in oligodendrogliomas. As the massive pre-treatments (radiotherapy, chemotherapy or PRRT) may increase the risk of secondary toxicity, especially in cases with pre-existing neurological deficits, the authors suggest to anticipate TAT and to use it in neo-adjuvant settings.

Notably, data on toxicity suggest that it is either negligible or limited to low grades, regardless of the radiopharmaceutical used. This holds true despite the limitations related to the small number of patients included in several of the studies reviewed. Similar numerical limitations apply to efficacy, although the data still suggest a potential for disease control.

Within the glioma literature, several aspects—most notably follow-up—remain insufficiently standardized and clearly defined. However, the reported CASP scores reflected an overall reviewers’ positive appraisal of the studies.

The innovative, pioneering character of this approach—predominantly oriented toward RLT rather than PRRT in gliomas—is underscored by the analysis of clinical trials, given that they:

- are all prospective and phase 1 or pilot study.
- tend to focus primarily on exploratory radiopharmaceuticals, or.
- rely on the use of established radiopharmaceuticals which, however, are usually employed in the treatment of other malignancies.

### Limitations

This review has some limitations.

First, despite the use of targeted keywords, a number of non-relevant articles still need to be excluded. Major scientific databases remain an essential resource, but they have certain limitations. These include automatic or partial indexing, semantic ambiguity of search terms, the lack of controlled vocabularies (e.g., MeSH, which are not implemented in Scopus and Web of Science), and the retrieval of articles that mention keywords only marginally, without substantively addressing the topic of interest. Notably, a marked difference emerged between studies on meningiomas and those on gliomas: Only 10.2% of the initially assessed meningioma studies met the inclusion criteria, compared with just 4.6% of glioma studies.

Second, remaining within the context of numerical differences, the uneven distribution of the studies—22 on meningiomas versus eight on gliomas—represents a limitation which could have had some impact on the overall balance and completeness of the evidence synthesis.

Finally, the heterogeneity of some parameters, such as study designs, radiopharmaceuticals, endpoints, times of follow-up, and patient populations, which limits the comparability across studies and precludes the possibility of performing a meta-analysis or draw definitive clinical recommendations.

## Conclusion

Overall, RLT emerges as a promising therapeutic approach in neuro-oncology. Its theranostic paradigm represents a distinctive advantage, enabling patient selection, treatment personalization and response monitoring. However, it is important to note that the current body of evidence is limited by the heterogeneity and small size of the studies reviewed, which significantly hampers the ability to generalize the findings. Therefore, future research should prioritize well-designed prospective and multicenter trials, standardized response criteria, and the exploration of novel radiopharmaceuticals (e.g., somatostatin receptor antagonists, PSMA ligands, and alpha-emitters). Such efforts may pave the way for future therapeutic applications in these challenging diseases.
